# Consequences of Chirality in Directing the Pathway of Cholesteric Helix Inversion of π‐Conjugated Polymers by Light

**DOI:** 10.1002/adma.202005720

**Published:** 2020-12-03

**Authors:** Chidambar Kulkarni, Rick H. N. Curvers, Ghislaine Vantomme, Dirk J. Broer, Anja R. A. Palmans, Stefan C. J. Meskers, E. W. Meijer

**Affiliations:** ^1^ Institute for Complex Molecular Systems and Laboratory of Macromolecular and Organic Chemistry Eindhoven University of Technology P.O. Box 513 Eindhoven 5600 MB The Netherlands; ^2^ Department of Chemistry Indian Institute of Technology (IIT) Bombay Powai Mumbai 400076 India; ^3^ Institute for Complex Molecular Systems and Laboratory for Functional Organic Materials and Devices (SFD) Eindhoven University of Technology P.O. Box 513 Eindhoven 5600 MB The Netherlands; ^4^ Institute for Complex Molecular Systems and Molecular Materials and Nanosystems Eindhoven University of Technology P.O. Box 513 Eindhoven 5600 MB The Netherlands

**Keywords:** azobenzene, chirality, cholesterics, fluorenes, helix inversion, photoswitches, supramolecular helicity

## Abstract

Control over main‐chain motion of chiral π‐conjugated polymers can lead to unexpected new functionalities. Here, it is shown that by combining photoswitchable azobenzene units in conjugation with chiral fluorene comonomers and appropriate plasticizers, the polymer organization and chiroptical properties of these alternating copolymers steered by light and its state of polarization can be dynamically controlled. The configuration of the stereogenic centers in the side chains of the fluorene units determines the handedness of the cholesteric organization in thermally annealed films, indicating cooperative behavior. The polymer alignment and helicity of the supramolecular arrangement can be switched by irradiating with linearly and circularly polarized light, respectively. Intriguingly, when switching the handedness of thermally induced cholesteric organizations by illuminating with circularly polarized light that is opposite to the handedness of the cholesteric phases, a nematic‐like intermediate state is observed during helix interconversion. By the sequence of irradiation with left and right circularly polarized light followed by thermal annealing, an asymmetric motion, reminiscent of that seen in molecular motors is observed. These findings suggest that functional conjugated polymers can exhibit emergent properties at mesoscopic scale.

## Graphical abstract

Alternating copolymers of fluorene and azobenzene exhibit exceptional chiroptical properties in annealed thin films. The chiroptical properties, polymer chain alignment, and their supramolecular helicity can be fully controlled by irradiating with polarized light. Intriguingly, the diastereomeric relationship between the supramolecular helicity of polymer and the circular polarization of light leads to an asymmetric helix inversion pathway, reminiscent of molecular motors.

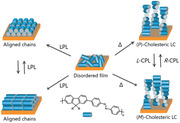

Controlling molecular motion at the nano‐, meso‐ and macroscopic length scale is actively pursued to create materials that can perform intricate functions.^[^ ^1^ ^]^ Such a control at single molecule level is achieved in molecular motors and ratchets.^[^ ^2^, ^3^, ^4^ ^]^ Liquid crystal networks are frequently used as materials to transform molecular deformations into macroscopic motion.^[^ ^5^, ^6^ ^]^ Facile alignment of mesogens (molecules exhibiting liquid crystalline phases) using polyimide layers is critical to observe the macroscopic motion. Similar strategies cannot be easily applied to functional, π‐conjugated polymers. In spite of the enormous progress,^[^ ^7^, ^8^ ^]^ it is a real challenge to dictate the organization of structurally rigid and functional polymers at mesoscopic levels in vitrified films and to translate or connect these (chiral) structures to macroscopic properties. Yet, such systems are required for applications in chiral‐selective data storage, steered polarized light emission, and directional macroscopic motion.

Light is a widely used stimulus to control molecular motion and organization through incorporation of photoactive units into molecular building blocks. Photomodulation of chiroptical properties is particularly attractive due to its potential applications in chiroptical switches,^[^ ^9^, ^10^, ^11^, ^12^, ^13^, ^14^, ^15^ ^]^ molecular motors^[^ ^16^ ^]^ and holography. Amongst the photoactive molecules,^[^ ^17^ ^]^ azobenzenes are widely studied to control a plethora of molecular properties.^[^ ^18^, ^19^, ^20^, ^21^, ^22^, ^23^, ^24^, ^25^, ^26^ ^]^ The well understood *trans*→*cis* and *cis*→*trans* isomerization of azobenzene is often used to modulate properties at the molecular level. Less known is the use of the oscillating character of the *trans*→*cis*→*trans* photoisomerization to reorganize polymeric chains at the meso‐ and macroscopic level. The continuous irradiation with polarized light reorganizes the molecules in such a way that the transition dipole moment of all azobenzenes are finally positioned perpendicular to the polarization of the incoming light; a phenomenon known for low molecular weight species. A rare phenomenon is the imprinting of chiral organization by circularly polarized light. Azobenzenes attached as side chains to covalent polymers have shown chiroptical switching.^[^ ^27^, ^28^, ^29^, ^30^, ^31^ ^]^ Whereas efficient photoswitching has often been observed in these materials, they lack the cooperative trigger to induce changes at a mesoscopic length scale due to: i) the low local density of photoactive molecules, ii) the often random organization and flexibility of the polymer main chain, and iii) the presence of flexible spacers between the mesogens (azobenzene) and the main chain. Azobenzenes in direct conjugation with an electronically active polymer backbones can lead to significant structural reorganization,^[^ ^32^, ^33^, ^34^, ^35^ ^]^ which offers the possibility of tuning the electronic properties of the polymer by employing the state of polarization of light. However, the photoswitchability in polymers containing azobenzene as part of the main‐chain is typically inefficient, particularly in thin film state due to the restricted motion of the often‐rigid polymer chains.

Previously, Scherf and co‐workers^[^ ^36^ ^]^ as well as our group^[^ ^37^ ^]^ have shown that poly(fluorene)s bearing enantiopure side chains exhibit exceptional chiroptical properties in annealed thin film with application in organic light‐emitting diodes. A recent review by Di Bari and co‐workers provides a comprehensive discussion on chiroptical properties of various poly(fluorene)s.^[^ ^38^ ^]^ Achiral, alternating copolymers of fluorene and azobenzene (poly(9,9′‐dialkylfluorene‐*alt*‐azobenzene) (PFAB)) have been reported with photocontrol over the properties of polymer films.^[^ ^33^, ^34^, ^39^ ^]^ Typically the photoswitching was found to be sluggish in thin film and sometimes the reverse isomerization (*cis*→*trans*) was not observed.^[^ ^34^ ^]^

Here, we report on the control over polymer organization and the mechanistic pathways of a cholesteric helix inversion of π‐conjugated polymers by polarized light. We study the supramolecular assembly of both enantiomeric forms of the alternating copolymer of fluorene and azobenzene with a fully π‐conjugated backbone. We are primarily interested in the manifestation of azobenzene isomerization on polymer reorganization at the mesoscopic level, where the function emerges, rather than studying the azobenzene photoisomerization process itself. Supramolecular plasticizers were used as a key element to facilitate polymer chain motion, and hereby enable reversible organization of the, otherwise too rigid, polymer chains. We begin by investigating the helical supramolecular organization of polymers (chiroptical properties) in annealed films and their modulation by unpolarized light. Then we show that by employing linearly polarized light, a cholesteric to nematic liquid crystalline (LC)‐ordering can reversibly modulated. In the last part of the manuscript, we present exciting kinetic observations of a molecular motor‐like motion in polymers at the mesoscopic level due to the diastereomeric relationship between the helical organization of polymers and the circularly polarized light used for irradiation.

The enantiopure PFAB polymers ((***S,S*)‐PFAB** and (***R,R*)‐PFAB**, **Figure** **1**a) were synthesized by standard Suzuki‐polycondensation and fully characterized by various techniques (see **Table** **1**; Scheme S1, Figures S1–S17, Supporting Information, for details). The phase behavior of (***S,S*)‐PFAB** was studied using differential scanning calorimetry (DSC) and polarized optical microscopy (POM). In the first heating run of DSC, (***S,S*)‐PFAB** showed a transition at ≈150 °C (see Figure S18, Supporting Information). POM studies showed that the polymer becomes more fluidic and the birefringence is increased when heated at 150 °C. The birefringence is retained after cooling to 20 °C (see Figure S19, Supporting Information). DSC and POM results together suggest a transition from an amorphous state to a vitrified liquid crystalline (LC) order of the polymer upon heating at 150 °C and subsequent cooling to room temperature. Similar phase behavior was observed for (***R,R*)‐PFAB** with a LC‐transition centered around 130 °C (Figures S18 and S20, Supporting Information). The difference in *T*_LC_ of the two enantiomeric polymers is proposed to be the result of the small difference in molecular weight (*M*_n_ of 12.1 and 10.1 kg mol^−1^ for (***S,S*)‐PFAB** and (***R,R*)‐PFAB**, respectively). The UV–vis spectra of spin‐coated thin film of (***S,S*)‐PFAB** showed a broad absorption in the range of 350–500 nm, attributed to the π–π* transition of the conjugated fluorene–azobenzene monomeric unit (see Figures S21 and S22, Supporting Information), in agreement with previous results on achiral analogues.^[^ ^33^, ^34^ ^]^ All the studies in this work were carried out on films spin‐coated on glass slides without any alignment layer, unless otherwise explicitly mentioned.

**Figure 1:**
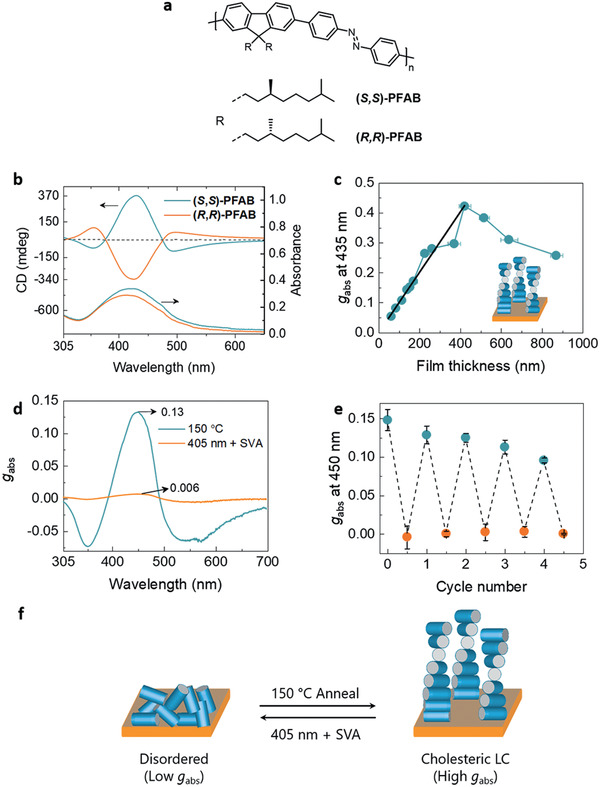
Chiroptical properties of thin films of poly(9,9′‐dialkylfluorene‐*alt*‐azobenzene) polymers and its modulation with depolarized light. a) Chemical structure of the PFAB polymers studied. b) CD and UV–vis spectra of annealed thin film (≈65 nm) of (***S*,*S*)‐PFAB** and (***R*,*R*)‐PFAB**. c) Thickness‐dependence of *g*_abs_ for (***S*,*S*)‐PFAB** polymer films. The error bars indicate the standard deviation in film thickness obtained by measuring thicknesses at multiple locations on the film. The solid black line is a guide to the eye to indicate a linear trend in *g*_abs_. The inset shows a schematic of cholesteric organization. d) CD spectra of the (***S*,*S*)‐PFAB** film when irradiated with depolarized light followed by thermal annealing at 150 °C. e) Changes in *g*_abs_ of (***S*,*S*)‐PFAB** obtained from CD spectra over multiple cycles of irradiation with depolarized light (orange data) followed by thermal annealing (blue data). The error bars represent the standard deviation from measurements on three independent samples. f) A schematic illustration of the change in polymer organization on irradiation with depolarized light and thermal annealing. The polymer chains are depicted as rigid rods. The CD studies in (d) and (e) were carried out on annealed films (≈60 nm) blended with 20 wt% of PEM‐OH as a plasticizer. Irradiation was carried out with 405 nm LED (27 ± 2 mW cm^−2^) in combination with SVA (dichloromethane as the solvent). All the spectra were recorded at 20 °C.

**Table 1:** Molecular and thermal properties of the polymers

Polymer	*M_n_* ^a)^ [kg mol^−1^]	*Đ* ^a^ ^)^	DP_n_ ^a^ ^)^	*T*_LC_ ^b)^ [°C]
(***S,S*)‐PFAB**	12.1	1.9	18	150
(***R,R*)‐PFAB**	10.1	1.7	15	130

^a)^ Determined from SEC measurements against polystyrene standards

^b)^ Obtained from the first heating run at 10 °C min^−1^ in DSC.

The circular dichroism (CD) spectra showed only a weak Cotton effect for spin‐coated films (see Figure S21a, Supporting Information). On thermal annealing of the film at 150 °C (*T*_LC_ of the polymer), a pronounced increase in CD was observed, with a maximum in dichroism coinciding with a maximum in absorbance (430 nm) (Figure 1b). By increasing the film‐thickness, the intensity of the CD enhanced remarkably (see Figure S23, Supporting Information). The dissymmetry factor for absorption of left and right circularly polarized light (*g*_abs_ = 2 × (*A*_L_ − *A*_R_)/(*A*_L_ + *A*_R_)) first shows a steady increase with film‐thickness (*d* < 400 nm) (Figure 1c). Then a maximum *g*_abs_ of +0.42 was observed for a film‐thickness of 420 nm. For film thicknesses beyond 420 nm, a drop in the *g*_abs_ (monitored at 430 nm) was observed. We attribute this drop in *g*_abs_ to the non‐uniform heat transfer to the polymer chains at the polymer–air interface during the annealing process, thus creating a gradient of polymer organization across the thickness rather than a uniform film. The large magnitude of *g*_abs_ and strong dependence of *g*_abs_ on film‐thickness for fluorene‐based polymers have been attributed to cholesteric LC‐ordering.^[^ ^40^, ^41^, ^42^ ^]^ We used Mueller‐matrix spectroscopy to study the organization of (***S,S*)‐PFAB** in aligned (by polyimide layer) and annealed films (Figures S24 and S25, Supporting Information).^[^ ^43^ ^]^ The results revealed that (***S,S*)‐PFAB** forms cholesteric LC‐ordering in annealed films with an estimated pitch of ≈1600 nm and adopts a right‐handed helical organization (*P*‐helicity). The enantiomeric counterpart (***R,R*)‐PFAB** showed similar film‐thickness dependence of *g*_abs_ (Figure S26, Supporting Information) and forms a left‐handed cholesteric organization (*M*‐helicity).

Initial attempts to modulate the cholesteric organization of (***S,S*)‐PFAB** by irradiating with a 405 nm LED showed minimal changes in both UV–vis absorption and CD spectra (see Figure S27, Supporting Information), indicating inefficient photoisomerization, due to the lack of main‐chain mobility in the vitrified films. To overcome this restricted polymer motion, we employed two known plasticizers. At room temperature, we used solvent vapor annealing (SVA), a well‐explored technique in the field of block‐copolymer self‐assembly to facilitate structural reorganization,^[^ ^44^ ^]^ and for elevated temperatures we used the additive polyethylene monoalcohol PEM‐OH (vide infra). Irradiation of a (***S,S*)‐PFAB** film with pseudo unpolarized 405 nm LED in the presence of dichloromethane vapor (SVA, Figure S28, Supporting Information) showed ≈20% decrease in the maximum absorbance. The corresponding CD signal decreased by ≈90% (see Figures S29 and S30, Supporting Information). These spectroscopic changes are compatible with a disordering brought about by reaching a photostationary state (with minor *cis*‐form) of azobenzenes in (***S,S*)‐PFAB**. The dichloromethane vapor locally swell the polymer chains, facilitating the continuously oscillating *trans→cis→trans* isomerization of azobenzenes in (***S,S*)‐PFAB**, which is sufficient to reduce the mesoscopic cholesteric LC‐ordering significantly. This result indicates that a small molecular deformation can trigger changes at the mesoscopic level due to cooperative effects. Since the azobenzene in (***S,S*)‐PFAB** is in direct conjugation with an electron donating fluorene moiety, it is conceivable that the UV–vis absorption peaks corresponding to n→π* and π→π* transitions of azobenzene in PFAB system are closer and may overlap to some extent, analogous to the properties of aminoazobenzene and pseudo‐stilbenes.^[^ ^25^, ^45^ ^]^ As a consequence, i) we do not observe the emergence of an absorption peak in the long wavelength region (450–500 nm) that is characteristic of n→π* transition of the *cis*‐isomer of azobenzene,^[^ ^24^ ^]^ and ii) irradiation of PFAB films with a 405 nm LED does not selectively excite the *trans*‐isomer of the azobenzene unit, thus accounting for reversibility of the photoconversion of the azobenzenes units.

Thermal annealing of films in the dark was undertaken to recover the cholesteric LC state. On thermal annealing (at 150 °C for 15 min without PEM‐OH), the CD intensity recovered to about half of its original (pre‐irradiation) value (see Figure S31, Supporting Information), indicating partial recover of cholesteric LC ordering. In line with our previous results,^[^ ^46^ ^]^ we observed that the addition of PEM‐OH enhances chain mobility of (***S,S*)‐PFAB** leading to improved chiroptical properties (Figure S32, Supporting Information). PEM‐OH blended films of (***S,S*)‐PFAB** showed complete recovery of CD intensity (*g*_abs_) (Figure 1d; Figure S33, Supporting Information), without influencing neither the photoisomerization nor the LC‐ordering (Figure S34, Supporting Information). Thus repeating the irradiation (405 nm, 27 ± 2 mW cm^−2^ with a depolarizer) in conjunction with SVA and thermal annealing (150 °C for 15 min) of PEM‐OH blended (***S,S*)‐PFAB** films, the strong chiroptical properties in films were reversibly modulated. The CD spectra showed negligible contribution from linear dichroism artifacts (Figure 1e; Figure S35, Supporting Information). Thus, by using depolarized light and thermal annealing, the polymer organization in the film was modulated between a disordered state with low *g*_abs_ and a cholesteric LC‐ordering with strong *g*_abs_ (Figure 1f).

Next, we explored the irradiation with linearly polarized light to photoalign the (***S,S*)‐PFAB** chains in thin film, analogous to that observed for conventional liquid crystals.^[^ ^45^ ^]^ Thermally annealed film of (***S,S*)‐PFAB** blended with 20 wt% PEM‐OH showed negligible linear dichroism (difference in absorption of light polarized along orthogonal directions) (**Figure** **2**a), indicating no preferred orientation of polymer chains in its cholesteric LC‐phase. On irradiating with linearly polarized light (405 nm with SVA), a significant increase in LD was observed, suggesting the photoalignment of polymer chains (Figure 2a). The LD arises from the alignment of the long axis of the polymer chains in a direction perpendicular to the polarization of the incident light.^[^ ^25^, ^45^ ^]^ Further irradiation with linearly polarized light with polarization direction orthogonal to the previous experiment gave an inversion in the sign of LD without significant change in the absolute magnitude (≈ 0.4) (Figure 2a). Such experiments repeated over multiple cycles showed no fatigue in the magnitude of LD (Figure 2b). The mechanism of photoalignment and photo‐reorientation of polymer chains can be attributed to a series of fast *trans*→*cis*→*trans* isomerizations, similar to that observed in azobenzene bearing LC polymers.^[^ ^45^ ^]^ To further confirm the anisotropic organization of polymer chains, static polarized UV–vis measurements were carried out. These studies showed highly anisotropic absorbance with a dichroic ratio (A_‖_/A_⊥_) of 5.05 and an order‐parameter of 0.57 at 430 nm (Figure S36, Supporting Information). Morphological characterization of a thermally annealed film by atomic force microscopy (AFM) showed no preferred orientation of polymer chains (Figure S37, Supporting Information). In contrast, AFM and POM image of a film irradiated with linearly polarized light showed aligned polymer chains both at shorter (1 µm) and larger (50 µm) length scale, respectively (see Figure 2c; Figure S38, Supporting Information). These results clearly suggest a reorganization of polymer chains such that the absorption of incoming polarization is minimized. In other words, polymer chains that can be visualized as rigid rods undergo a reversible 90° rotation on irradiation with linearly polarized light (Figure 2d). As a result, they lose their cholesteric LC‐ordering and mimic a nematic LC‐ordering.

**Figure 2:**
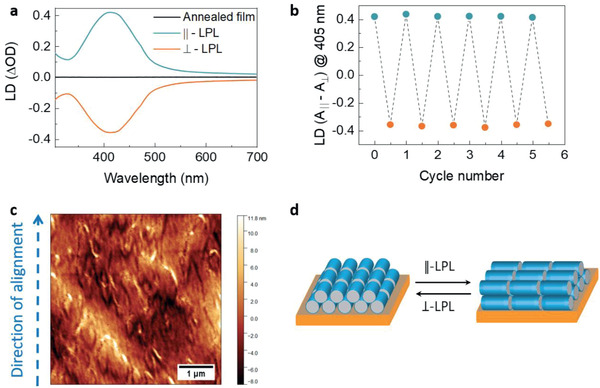
Effect of linearly polarized light on polymer organization. a) Linear dichroism spectra of (***S*,*S*)‐PFAB** when irradiated with linearly polarized light while maintaining the sample orientation. b) Variation of linear dichroism over multiple cycles of irradiation with linearly polarized light either parallel or perpendicular to an arbitrary direction in the plane of the film. c) Tapping mode AFM image of (***S*,*S*)‐PFAB** film after irradiation with 405 nm linearly polarized light in presence of SVA. The film thickness is ≈70 nm. d) A schematic showing the reversible reorganization of polymer chains (depicted as rods) on irradiation with linearly polarized light. The irradiation is with respect to an arbitrary chosen vertical axis. The LD measurements ((a) and (b)) were carried out on annealed films (≈60 nm) blended with 20 wt% of PEM‐OH as a plasticizer. Irradiation was carried out with 405 nm LED (10 ± 1 mW cm^−2^) in combination with SVA (dichloromethane as the solvent). All the spectra are recorded at 20 °C.

In the next step, we investigated the control of the helicity of the cholesteric organization (*P* or *M*) by irradiating the thin films with circularly polarized light (CPL). Thermally annealed films of (***S,S*)‐PFAB** and (***R,R*)‐PFAB** have *P*‐ and *M*‐helicity, respectively (vide supra). Irradiation of a 20 wt% PEM‐OH blended (***S,S*)‐PFAB** film with *L*‐CPL and *R*‐CPL in conjunction with SVA showed a negative and positive CD signal at 430 nm, respectively (**Figure** **3**a). This suggests that the helicity of cholesteric polymer organization is reversed on irradiation with CPL. By alternatingly irradiating with *R*‐ and *L*‐CPL, the helicity of polymer organization was reversibly modulated over multiple cycles (Figure 3b). It is to be noted that the magnitude of the CD signal is unequal when irradiated with *R*‐ and *L*‐CPL. This might arise due to the diastereomeric interaction of enantiopure (***S,S*)‐PFAB** with incident *R*‐ and *L*‐CPL, leading to induced *P*‐ and *M*‐helical organizations of the polymer which are also diastereomerically related with one another.

**Figure 3:**
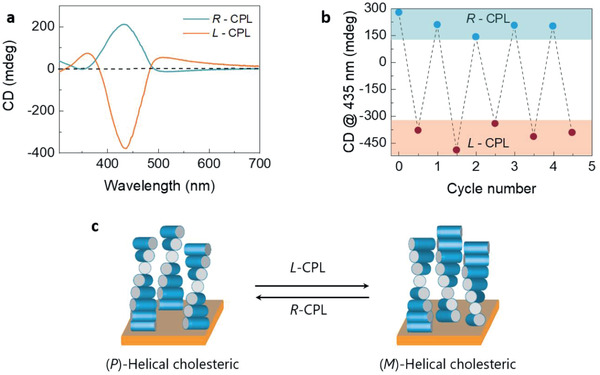
Effect of circularly polarized light on polymer organization. a) Circular dichroism spectra of (***S*,*S*)‐PFAB** when irradiated with *R*‐ and *L*‐CPL. b) Changes in CD effect over multiple cycles of irradiation with CPL. c) A schematic illustrating the change in helicity of cholesteric organization on irradiation with CPL. The polymer chains are depicted as rigid rods. The studies were carried out on annealed films (≈60 nm) blended with 20wt% of PEM‐OH as a plasticizer. Irradiation was carried out with 405 nm LED (5 ± 1 mW cm^−2^) in combination with SVA (dichloromethane as the solvent). All the spectra are recorded at 20 °C.

Finally, we performed in‐situ kinetic measurements on films of PFAB polymers to evaluate the effect of chirality of PFAB polymers on the helicity switching (*P↔M*) brought about by CPL. For kinetic measurements, a modified CPL spectrometer set‐up was used (see Figure S39, Supporting Information for detailed experimental set‐up). In contrast to typical kinetic experiments where a given system is perturbed only once using an external agent (light, heat, additive, etc.,) and the corresponding response is measured immediately thereafter, here we studied the time evolution of the spectroscopic signature while the polymer film was being continuously irradiated with CPL. Purely *L*‐CPL and *R*‐CPL have dissymmetry factor (*g*‐value) of −2 and +2, respectively. For in‐situ kinetic measurements, a film of PFAB polymer was continuously irradiated with CPL (either *R* or *L*) and the variation in the dissymmetry factor of the transmitted CPL (termed “*g*_trans‐CPL_”) was recorded. It is to be noted that *g*_abs_ signifies the differential absorption of the CPL by the polymer film, whereas the *g*_trans‐CPL_ describes the dissymmetry factor for the transmitted CPL after interaction with the polymer film. Since an annealed PFAB polymer film is cholesterically ordered (with an appreciable *g*_abs_), the incident CPL interacts with the polymer film and as a result the magnitude of the transmitted CPL (*g*_trans‐CPL_) is altered. In other words, the changes in the *g*_trans‐CPL_ are a measure of the interaction between the polymer film helicity and the irradiated CPL.

When a thermally annealed film of (***S,S*)‐PFAB** having (*P*)‐helicity (denoted as (*P*)_T_, with subscript “T” denoting thermally favored state) was irradiated with *L*‐CPL, the kinetic profile shows a damped oscillatory behavior in *g*_trans‐CPL_ with the final state being (*M*)‐helicity (denoted as (*M*)_Ph_, with subscript “Ph” denoting the photofavored state) (**Figure** **4**a; Figure S40, Supporting Information). Subsequently switching the polarization of irradiation to *R*‐CPL, we observe an exponential depolarization profile (Figure 4b), suggesting a (*M*)_Ph_ → (*P*)_Ph_ helicity change. The variation of *g*_trans‐CPL_ by irradiation with *L*‐CPL and *R*‐CPL do not overlap, indicating that (*P*)_T_ → (*M*)_Ph_ and (*M*)_Ph_ → (*P*)_Ph_ helicity changes occurs through different pathways. It is to be noted that thermal annealing of (*P*)_Ph_ state converts it into its thermally favored (*P*)_T_‐helicity (vide supra), thus completing a full cycle (Figure 4c). Similar studies on (***R,R*)‐PFAB** also showed different pathways for helicity interconversion between (*M*)_T_ → (*P*)_Ph_ and (*P*)_Ph_ → (*M*)_Ph_ (Figure S41, Supporting Information), indicating that the absolute configuration of the stereogenic centers in the side chains of the polymer influence the pathways of supramolecular helicity change.

**Figure 4:**
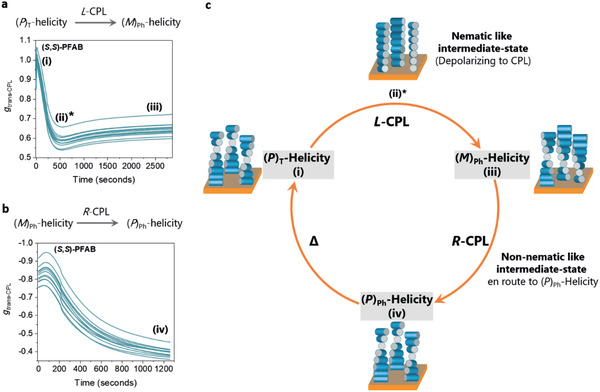
Kinetics of in situ helicity switching (*P*↔*M*) of **(*S,S*)‐PFAB** films of 130 nm thickness. All the studies were carried out under SVA with irradiation by 455 nm LED (5 ± 1 mW cm^−2^). a) The time evolution of *g*_trans‐CPL_ when a thermally annealed film of (***S*,*S*)‐PFAB** ((*P*)_T_‐helicity) was continuously irradiated with *L*‐CPL (a) followed by *R*‐CPL (b). The experiments in (a) and (b) were performed sequentially on the same polymer film. The change in the sign of the *g*_trans‐CPL_ (*Y*‐axis) in (a) and (b) is due to the switch in irradiation from *L*‐ to *R*‐CPL. The various traces in (a) and (b) represent different monitoring wavelengths between 450–460 nm. c) A schematic illustration of the change in supramolecular helicity on irradiation of (***S*,*S*)‐PFAB** ((*P*)_T_‐helicity) with *L*‐ and *R*‐CPL. The (*P*)_Ph_‐helicity on thermal annealing at 150 °C (denoted by “Δ”) reverts back to (*P*)_T_‐helicity, thus completing a full cycle. The polymer chains are represented by rigid rods. The state marked with an asterisk (*) indicates an intermediate, nematic‐like state.

We note that the oscillatory kinetic profiles are sensitive to various factors such as film‐thickness (Figure S42, Supporting Information), and polymer film history in terms of helicity. Full understanding of such a fascinating phenomenon requires a more detailed study. However, a strong depolarization of CPL is observed in the oscillatory profiles (states marked with an asterisk in Figure 4a) for the (*P*)_T_ → (*M*)_Ph_ helicity conversion for (***S,S*)‐PFAB** and (*M*)_T_ → (*P*)_Ph_ helicity conversion for (***R,R*)‐PFAB** (Figure S41a, Supporting Information). It is intuitive to envisage that the supramolecular helicity interconversion on irradiating with CPL involves a gradual change in the twist angle between any two consecutive layers of polymer chains. In this process, a state can be achieved in which the polymer chains have very low twist angle, or in other words the polymer chains are almost parallel to each other (a nematic‐like arrangement). Such a nematic intermediate is expected to be strongly depolarizing to the incoming CPL and thus accounts for the changes observed in Figure 4a and Figure S41a, Supporting Information. A strikingly similar nematic intermediate state is observed for light (unpolarized) induced helicity interconversion in small molecule based cholesteric liquid crystals,^[^ ^47^, ^48^ ^]^ suggesting that functional conjugated polymers (PFAB) can display characteristic features of small molecule based liquid crystals. In addition, irradiating with circularly polarized light on a chiral polymer film has important consequences. The asymmetry at the molecular level (chirality) for **(*S,S* )**‐ and (***R,R*)‐PFAB** polymers in combination with the polarization of CPL irradiation and thermal annealing lead to one complete cycle of helicity interconversion for, example, (*P*)_T_ → (*M*)_Ph_ → (*P*)_Ph_ → (*P*)_T_ (Figure 4c). Each step of the helicity interconversion cycle goes through a unique intermediate state at the mesoscopic level, reminiscent of a molecular motor.

In summary, we have shown that both enantiomers of the alternating copolymers of fluorene and photoswitchable azobenzene form cholesteric liquid crystalline phases in annealed thin films with strong chiroptical properties. Such properties of the mesophases in thin film were reversibly modulated by light and thermal annealing. Plasticizers such as solvent vapor and long chain alcohol (PEM‐OH) were key ingredients to observe reversible switching of chiroptical properties. By using linearly or circularly polarized light, the organization and supramolecular helicity of polymer chains in films were reversibly modulated due to the oscillating *trans→cis→trans* isomerization of the azobenzene units. The mechanism of *in‐situ* switching of supramolecular helicity (*P↔M*) on continuous irradiation with CPL shows an asymmetry. Although it is intuitive to envisage that chirality at the molecular level translates to asymmetry in the pathways of supramolecular helicity interconversion, such a phenomenon has not been explicitly demonstrated in systems other than molecular motors. The diastereomeric relationship between the supramolecular helicity of PFAB polymers and the chirality of CPL is the key determining factor in observing a molecular motor like behavior at the mesoscopic level. Finally, the fundamental insights obtained into controlling the helical organization of enantiopure conjugated polymers by light is envisaged to act as a testbed for the contemporary effort towards harnessing molecular chirality in electronic and spintronic devices.

## Conflict of Interest

The authors declare no conflict of interest.

## Supporting information

Supporting Information
