# Supplementary material for: Consequences of Chirality in Directing the Pathway of Cholesteric Helix Inversion of π‐Conjugated Polymers by Light
Source: Adv Mater. 2020 Dec 3;33(2):2005720. doi: 10.1002/adma.202005720 (PMC11468155; doi:10.1002/adma.202005720)
Supplement: Supplementary file 1 — Supporting Information [file ADMA-33-2005720-s001.pdf]

© 2020 Wiley-VCH GmbH

ADVANCED MATERIALS

Supporting Information

for *Adv. Mater.*, DOI: 10.1002/adma.202005720

Consequences of Chirality in Directing the Pathway of Cholesteric Helix Inversion of #-Conjugated Polymers by Light

Chidambar Kulkarni, Rick H. N. Curvers, Ghislaine Vantomme, Dirk J. Broer, Anja R. A. Palmans, Stefan C. J. Meskers,* and E. W. Meijer**

Copyright Wiley-VCH GmbH, 2020.

Supporting Information

Consequences of Chirality in Directing the Pathway of Cholesteric Helix Inversion of π-Conjugated Polymers by Light

Chidambar Kulkarni,[1,2,] Rick H. N. Curvers,[1] Ghislaine Vantomme,[1] Dirk J. Broer,[3] Anja R. A. Palmans,[1] Stefan C. J. Meskers,[4,]* and E. W. Meijer[1,]**

1. Institute for Complex Molecular Systems and Laboratory of Macromolecular and Organic Chemistry, Eindhoven University of Technology, P.O. Box 513, 5600 MB Eindhoven, The Netherlands,
   Email: e.w.meijer@tue.nl and chidambark@chem.iitb.ac.in

2. Department of Chemistry, Indian Institute of Technology (IIT) Bombay, Powai, Mumbai – 400076, India

3. Institute for Complex Molecular Systems and Laboratory for Functional Organic Materials and Devices (SFD), Eindhoven University of Technology, P.O. Box 513, 5600 MB, Eindhoven, The Netherlands

4. Institute for Complex Molecular Systems and Molecular Materials and Nanosystems, Eindhoven University of Technology, P.O. Box 513, 5600 MB Eindhoven, The Netherlands
   Email: s.c.j.meskers@tue.nl

Table of Contents:

1. Materials and Methods
2. Synthesis and molecular characterization
3. Supplementary figures
4. References

1. Materials and Methods:

All reagents were purchased from Sigma-Aldrich, Acros Organics, TCI, and Merck. Starting materials (*S*)-(-)-β-Citronellol (purity > 99% and 98.4% *ee*) and (*R*)-(+)- β-Citronellol (purity > 97% and 99% *ee*) were purchased from Takasago and Sigma-Aldrich, respectively.[1] The reagents were used as received unless otherwise stated. All solvents were purchased from Biosolve. Deuterated solvents were purchased from Cambridge Isotopes Laboratories. Reactions were monitored by thin-layer chromatography (TLC) using Silica-gel 60 F254 TLC-plates from Merck. TLC plates were visualized using either cerium molybdate or iodine stain. Automated column chromatography was performed on a Biotage Isolera™ One system using Biotage or Grace Reveleris® silica flash cartridges.

NMR spectra were recorded using a Bruker UltraShield NMR spectrometer operated at 400 MHz for ^{1}H and 100 MHz for ^{13}C nuclei. Chemical shifts are reported in ppm downfield from trimethylsilane (TMS). Peak multiplicity is abbreviated as: singlet (s), doublet (d), triplet (t), doublet of doublets (dd), and multiplet (m).

Infrared (IR) spectroscopy was performed on a Perkin Elmer FT-IR Spectrum Two spectrometer.

Matrix assisted laser desorption ionization time-of-flight mass spectrometry (MALDI-TOF-MS) was performed on a Bruker Autoflex Spectrometer using α-cyano-4-hydroxycinnamic acid (CHCA) and *trans*-2-[3-(4-tert-butylphenyl)-2-methyl-2-propenylidene]malononitrile (DCTB) as the matrices. Samples were prepared in chloroform with a concentration of roughly 1 mg/mL.

Gas Chromatography Mass Spectrometry (GC-MS) measurements were performed on a Shimadzu GC-17A gas chromatograph with a Shimadzu AOC-20i autoinjector, Shimadzu GCMS-QP2010 Plus mass spectrometer and a Phenomenex Zebron ZB-5MS column (l= 30 m, I.D. = 0.25 mm, thickness (0.25 μm). The temperature profile for the GC-MS measurements was 1-minute isothermal at 80 °C, followed by heating at 30 °C/mins to 300 °C and then an isothermal stage at 300 °C.

Elemental analysis was performed using a Perkin Elmer 2400 Series II CHNS/O Analyzer equipped with a Perkin Elmer AD-4 Autobalance.

Size exclusion chromatography (SEC) was performed on a Shimadzu Prominence-i LC-2030C 3D equipped with a RID-20A detector and polystyrene as the calibration standard. Samples were prepared in THF and the solutions were filtered through a 0.2 μm PTFE filter.

Thermal stability of the polymers in air and nitrogen atmosphere was assessed by thermogravimetric analysis (TGA) using a TA Instruments TGA Q500 at a rate of 10 °C/min.

Differential scanning calorimetry (DSC) was performed on a TA Instruments DSC Q2000 with indium as a standard. T_{LC} were taken at the midpoint of their respective transition.
Polarized optical microscopy was carried out using a Nikon Eclipse Ci POL equipped with a Linkam LTS 420 heating stage.
CD spectra were measured on a JASCO J-815 CD Spectrometer equipped with a Peltier temperature controller. A scanning rate of 100 nm/min, a bandwidth of 2 nm, a response time of 0.5 s, a data pitch of 0.1 nm, and single accumulation were employed. Baseline measurements were recorded using the same parameters for a cleaned glass slide. The g_{abs} was calculated by [ellipticity (mdeg)]/[32,980 × absorbance] at a particular wavelength.
UV-vis transmission spectra were recorded on a Perkin Elmer Lambda 1050 UV-vis-NIR spectrophotometer. A scanning rate of 141.20 nm/min and a 0.5 nm data interval were employed. Baseline correction was carried out by measuring the transmission of cleaned glass slides without polymer layer.
The film thickness was determined by using a Veeco Dektak 150 Surface Profiler.
Polarized transmission and reflection measurements were carried out using a Woollam WVASE ellipsometer using the general Mueller matrix measurement protocol.

Light irradiation was performed using a Thorlabs DC4104 advanced four-channel LED driver equipped with a DC4100-HUB. The LED driver was employed under constant current operation. The intensity of light was measured using Radiometer RM-12.

Polarized UV-vis studies were carried out on Perkin-Elmer Lambda 950 spectrometer with polarization accessory.

Atomic force microscopy (AFM) was carried out on Asylum Research MFP-3D mounted on an anti-vibration stage. AFM was performed in tapping-mode using Silicon probes manufactured by NanosensorsTM (model PPP-NCSTR-50) with a tip height of 10-15 μm and radius of <10 nm. Resolution of 512 × 512 pixel and a scan rate of 0.8 Hz was used for image acquisition. The images were subjected to first order flattening using Gwyddion (v. 2.48) software.

Density functional theory (DFT) based computations were carried out using Gaussian-09 suite of programs.[2] B3LYP exchange correlation functional,[3,4] 6-31+G(d,p) basis set and "int=ultrafine" keyword was used for the computations. All the computations were carried out

on a model compound of the repeat unit in which the alkyl chains were replaced by methyl groups for computational tractability. Frequency calculation on the optimized geometry showed no imaginary frequencies, confirming that the geometry is at least a local minimum, if not the global minimum. Time-dependent DFT (TD-DFT) computations were carried out considering 36 excitations (key word “nstates”) on the optimized ground state geometry to obtain the UV-vis spectra of the *trans*-form of the repeat unit.

Thin film preparation and annealing

A solution of PFAB polymer in chloroform was heated at 70 °C in an oil bath for 1 – 2 h and within 30 min after cooling down to room temperature, the solution was spin-coated on clean glass slides. Prior to spin-coating, all glass slides were cleaned by sonication with isopropyl alcohol (2x), and acetone (2x). Annealing of all thin film was carried out in a glovebox with a nitrogen atmosphere. To obtain thin film of different thickness, the concentration of the polymer solution was varied in between 10 – 105 mg/mL, and the spin speed was varied in between 800 – 4000 rpm. Three lines were scratched in the glass slides and the local thickness of two different spots on each line was determined. The overall thickness of the polymer layer on the glass slide was taken as the average of the six measurements. All prepared thin film were stored in the dark and protected by aluminum foil.

Preparation of polyimide rubbed thin film:

Glass slides were cleaned by sonication with isopropyl alcohol (2x), and acetone (2x). These glass slides were then etched in a UV-ozone photoreactor (PR-100) for 30 min. Planar polyimide solution (Merck AL 1051) was spin-coated on the etched glass slides (5000 rpm, 40 s, 500 rpm acceleration), and then annealed at 180 °C for 90 min. The polyimide layer was rubbed on a velvet cloth to induce planar alignment. A solution of **(*S*,*S*)-PFAB** in chloroform was heated to 70 °C for 1 – 2 h and within 30 min after cooling down to room temperature was spin-coated on cleaned glass slides and rubbed polyimide coated glass slides. Annealing of all thin film was carried out at 150 °C for 15 min in a glovebox under a nitrogen atmosphere. To obtain thin film of different thickness, the concentration of the polymer solution was varied between 10 – 105 mg/mL, and the spin speed was either 1200 or 1500 rpm. The thickness of thin film on PI aligned glass slides was assumed to be the same as the thickness of films obtained under similar conditions on an unaligned glass slide. All prepared thin film were stored in the dark and protected by aluminum foil.

Photo-irradiation of thin films:

Thin film of PFAB polymer were placed in a jar filled with roughly 50 mL DCM with the polymer layer facing in the upward direction, such that the films were not in direct contact with the solvent. This jar was closed with a quartz lid to ensure a saturated environment of solvent vapors (Figure S13). The thin film were irradiated by a 405 nm LED light source operated in a constant current mode (650 mA) for 15 minutes. After this time, the film was taken out and CD studies were performed at 20 °C. Following additional methods were employed to control the polarization of the incident light from the LED.

1. Pseudo-unpolarized (depolarized) light: We observed that the light directly incident from the LED had some residual linear polarization. Thus, to achieve pseudo-unpolarized light we have employed a depolarizer (From Thorlab DPU-25-A). Thus, a depolarizer was inserted between the LED and the jar containing the film for experiments with unpolarized light. The intensity of LED light on sample with the above configuration is 27±2 mW/cm^2.
2. Linearly polarized light (LPL): To achieve LPL, an adjustable linear polarizer was used between the LED and the jar containing the film. The intensity of LED light using a linear polarizer is 10±1 mW/cm^2.
3. Circularly polarized light (CPL): CPL was achieved by adjusting the linear polarizer at 45° and 315° with respect to the optical axis of a Fresnel rhomb. The handedness of the CPL was confirmed by a polarizer with the known CPL. The intensity of LED light using a linear polarizer and a Fresnel rhomb is 5±1 mW/cm^2.

2. Synthesis and molecular characterization

Since the stereogenic centers in citronellol (either (*S*)- or (*R*)-configuration) do not take part in any chemical transformations on the citronellol, we assume that the resultant polymers (***(S,S)*-PFAB** and ***(R,R)*-PFAB**) bearing these side chains also have high optical purity similar to the citronellol starting materials. The monomers 2,7-bis(pinacoyl)-9,9-di[(*S*)-3,7-dimethyloctyl]fluorene bisboronic ester (**BPin-F-BPin**)[5] and 1,2-bis(4-bromophenyl)diazene (**Br-AB-Br**)[6] were synthesized and characterized according to literature procedures. Synthesis of **BPin-F-BPin** was carried out with 1,4-dioxane as the solvent, instead of DMF and recrystallized from ethanol. The purity of the monomers was confirmed through their sharp melting point. The synthesis of ***(S,S)*-PFAB and *(R,R)*-PFAB** was carried out according standard Suzuki polycondensation methods.[5]

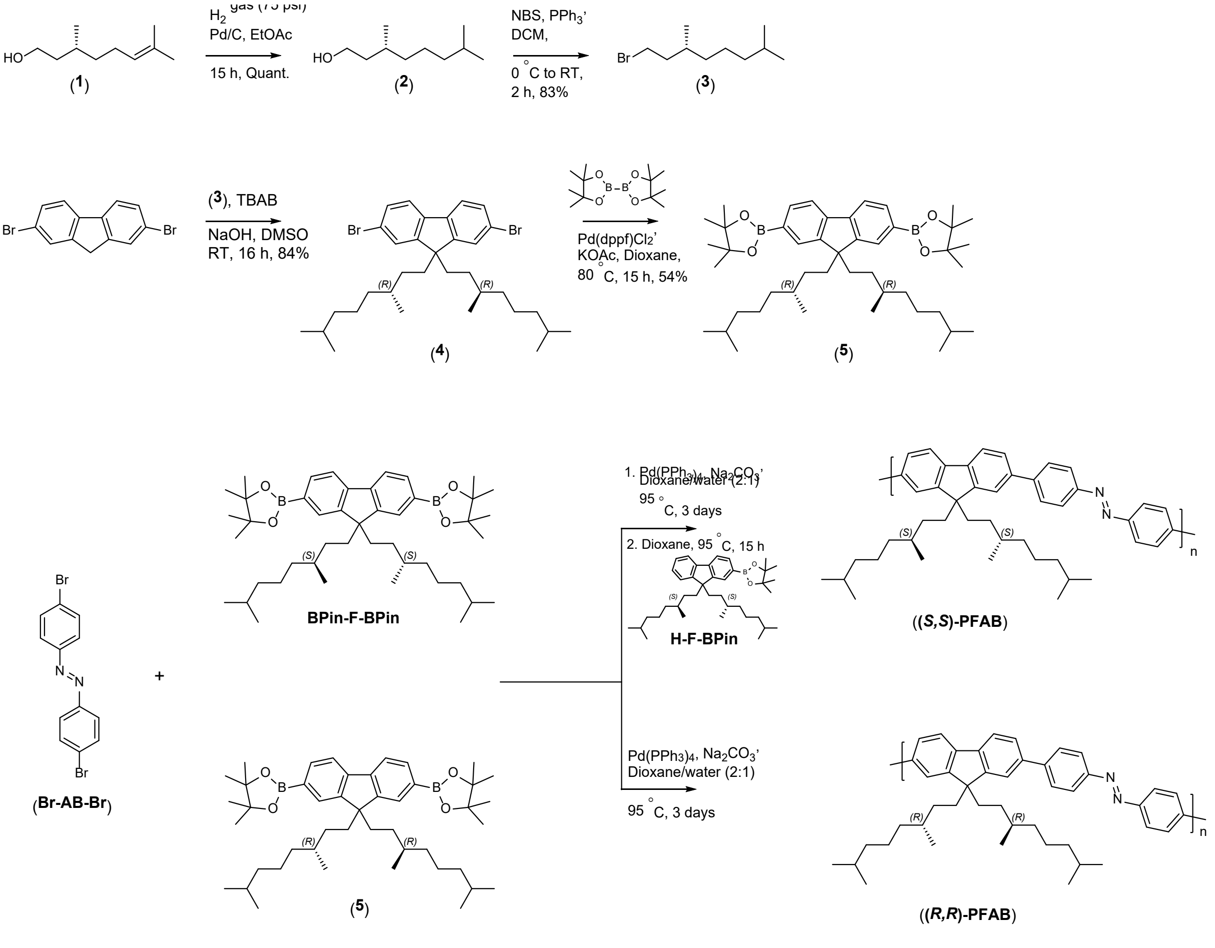

Scheme 1: Synthetic route to ***(S,S)*-PFAB** and ***(R,R)*-PFAB** polymers.

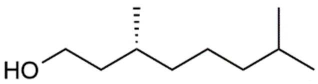

Synthesis of (*R*)-3,7-dimethyloctan-1-ol (2): (*R*)-3,7-dimethyloct-6-en-1-ol (**1**, 5 g, 32 mmol) was added to a Parr reactor vessel (250 mL) and off the shelf ethyl acetate (70 mL) was added. The solution of **1** in ethyl acetate was

degassed by bubbling argon for 30 mins. Immediately after, activated Pd/C catalyst (140 mg) was added to the vessel. The reaction was carried out in a shaker hydrogenation apparatus (Parr-reactor) with continuous shaking. The hydrogen gas pressure was set to 75 psi, as the reaction progressed a steady drop in the pressure was observed, indicating the consumption of the hydrogen gas. The reaction was carried out until no further drop in hydrogen pressure was observed (15 hours). Then the reaction mixture was filtered over a pad of Celite (diatomaceous earth) and the residue was thoroughly washed with ethyl acetate. The collected ethyl acetate was evaporated to obtain **2** as a clear oil (5.0 g, quantitative yield). The completion of the reaction was confirmed by GC-MS.

^{1}H-NMR (400 MHz, $CDCl_3$): δ 3.73 – 3.62 (m, 2H), 1.64 – 1.47 (m, 3H), 1.41 – 1.35 (m, 1H), 1.33 – 1.06 (m, 7H), 0.89 (d, *J* = 6.6 Hz, 3H), 0.86 (d, *J* = 6.6 Hz, 6H) ppm.

^{13}C-NMR (100 MHz, $CDCl_3$): δ 61.41, 40.14, 39.39, 27.50, 29.64, 28.11, 24.82, 22.83, 22.73, 19.78 ppm.

GC-MS: R_t 3.65 mins

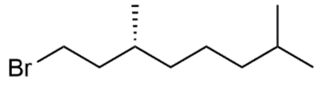

Synthesis of (*R*)-1-bromo-3,7-dimethyloctane (3): (*R*)-3,7-dimethyloctan-1-ol (**2**) (4.04 g, 25.5 mmol, 1.0 eq.) and triphenylphosphine (5.62 g, 31.5 mmol, 1.2 eq.) were added to a round bottom flask (250 mL). Off the shelf dichloromethane (100 mL) was added to the RBF and the flask was cooled to 0 °C on an ice-bath. *N*-Bromosuccinimide (8.26 g, 31.5 mmol, 1.2 eq.) was added in portions to the RBF under constant stirring. After complete addition, the ice-bath was removed and the slightly pale-yellow reaction mixture was stirred at room temperature under argon atmosphere for 2 hours. The progress of the reaction was monitored by GC-MS. After the reaction was completed, the contents of the flask were evaporated to obtain a slight yellow solid. To this, excess *n*-hexane was added, sonicated and filtered through a glass funnel over a pad of silica-gel (60 Å, 230 – 400 mesh). The filtrate was concentrated on a rotary evaporator to obtain **3** as a clear liquid (4.66 g, 83% yield).

^{1}H-NMR (400 MHz, $CDCl_3$): δ 3.49 – 3.37 (m, 2H), 1.92 – 1.82 (m, 1H), 1.71 – 1.58 (m, 2H), 1.55 – 1.47 (m 1H), 1.32 – 1.21 (m, 3H), 1.18 – 1.09 (m, 3H), 0.89 – 0.86 (m, 9H) ppm.

^{13}C-NMR (100 MHz, $CDCl_3$): δ 40.22, 39.31, 36.85, 32.39, 31.81, 28.09, 24.68, 22.82, 22.73, 19.10 ppm.

GC-MS: R_t 4.02 mins

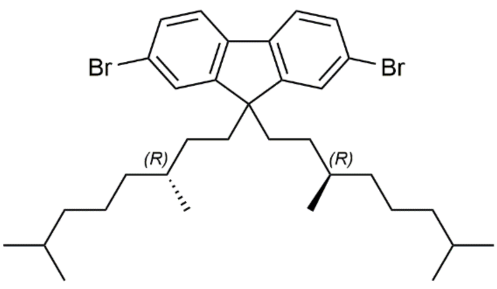

Synthesis of 2,7-dibromo-9,9-bis((*R*)-3,7-dimethyloctyl)-9*H*-fluorene (4): Sodium hydroxide (3 g) and deionized water (5 mL) were added to a 3-necked RBF (250 mL) and stirred at room temperature. Off the shelf dimethyl sulfoxide (80 mL) was added to the RBF and stirred at room temperature for 15 minutes. Then 2,7-dibromofluorene (2.96 g, 9.16 mmol, 1.0 eq.) and tetrabutylammonium bromide (220 mg, 0.68 mmol, 0.07 eq.) were added to the RBF and stirred under argon atmosphere at room temperature for 30 minutes. On addition of 2,7-dibromofluorene the solution turned orange and over the course of 30 minutes it turned dark purple. Compound **3** (4.66 g, 21 mmol, 2.3 eq.) dissolved in dimethyl sulfoxide (20 mL) was added to the RBF and the reaction mixture was stirred at room temperature. After 15 hours, the reaction mixture was diluted with DCM and transferred to a separating funnel. Deionized water (100 mL) was added to the separating funnel. The organic layer was extracted with 1 M HCl (2 × 100 mL) and finally with deionized water (2 × 100 mL). The collected organic layer was dried over sodium sulfate and evaporated using a rotary evaporator to obtain a dark oil. To this, potassium tetrabutoxide (2.88 g) and dry THF (50 mL) were added and the suspension was stirred at room temperature. After 3.5 hours the solution was filtered through a sintered glass funnel and the solids were washed with excess THF. The filtrate was concentrated under vacuum to obtain a dark oil. This was dissolved in excess *n*-heptane by sonication and filtered. The filtrate was evaporated under reduced pressure to obtain a dark colored oil (5.3 g of crude product). The crude product was purified by column chromatography on a Biotage Isolera® system with SNAP-KP-SIL (100 g) cartridge and eluting with *n*-heptane. Compound **4** eluted as a UV-active product within a few column volumes and was obtained as a clear oily product (4.3 g, 84% yield). The oily product solidifies on standing at -20 °C for a few days.

^{1}H NMR (400 MHz, $CDCl_3$): δ 7.51 (d, *J* = 8 Hz, 2H), 7.46-7.43 (m, 4H), 1.99-1.85 (m, 4H), 1.49-1.40 (m, 2H), 1.3-1.25 (m, 2H), 1.14-0.96 (m, 12H), 0.81 (d, *J* = 6.64 Hz, 12H), 0.69 (d, *J* = 6.52 Hz, 6H), 0.60-0.51 (m, 2H), 0.47-0.38 (m, 2H) ppm.

^{13}C-NMR (100 MHz, $CDCl_3$): δ 152.63, 139.28, 130.29, 126.25, 121.61, 121.25, 55.65, 39.30, 37.56, 36.65, 32.91, 30.45, 28.07, 24.69, 22.82, 22.73, 19.59 ppm.

MALDI-TOF MS: m/z calculated for $[C_{33}H_{48}Br_2]^+$ 602.2122, found 602.30 $[M]^{\cdot+}$.

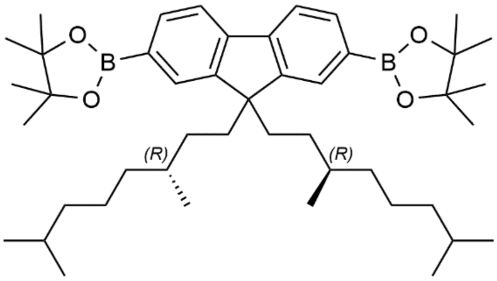

Synthesis of 2,2'-(9,9-bis((*R*)-3,7-dimethyloctyl)-9*H*-fluorene-2,7-diyl)bis(4,4,5,5-tetramethyl-1,3,2-dioxaborolane) (5): Compound **4** (1.96 g, 3.24 mmol, 1.0 eq.), bis(pinacolato)diboron (2.47 g, 9.72 mmol, 3.0 eq.), potassium acetate (1.90 g, 19.44 mmol, 6.0 eq.) and Pd(dppf)Cl_2 (160 mg, 0.21 mmol, 0.06 eq.) were added to a 3-necked round bottom flask, which was evacuated and back-filled with nitrogen (3 times). Degassed dioxane (by 3 cycles of freeze-pump-thaw, 35 mL) was added to the RBF and the reaction mixture was heated at 80 °C for 15 hours. Then the reaction mixture was cooled down to room temperature and the contents of the flask were reduced under vacuum. The dark residue was redissolved in DCM and transferred to a separating funnel. The organic layer was extracted with water and brine. The collected organic layer was dried over sodium sulfate and evaporated under reduced pressure to obtain a dark semi-solid crude product. The crude product was purified by column chromatography eluting with 20 – 70% DCM in *n*-heptane. The desired fraction containing the product was evaporated, and further purified by recrystallization from hot ethanol. This afforded compound **5** as white needle-like crystals (1.22 g, 54% yield).

^{1}H NMR (400 MHz, $CDCl_3$): δ 7.80 (d, *J* = 7.52 Hz, 2H), 7.74 – 7.70 (m, 4H), 2.07 – 1.93 (m, 2H), 1.45 – 1.37 (m, 26H), 1.08 – 0.93 (m, 12H), 0.78 (d, *J* = 6.56 Hz, 12H), 0.64 (d, *J* = 6.44 Hz, 6H), 0.57 – 0.48 (m, 2H), 0.44 – 0.35 (m, 2H) ppm.

^{13}C-NMR (100 MHz, $CDCl_3$): δ 150.54, 144.13, 133.77, 129.01, 119.48, 83.80, 55.13, 39.31, 37.48, 36.67, 33.00, 30.48, 28.02, 25.05, 24.69, 22.84, 22.74, 19.65 ppm.

MALDI-TOF MS: m/z calculated for $[C_{45}H_{72}B_2O_4]^+$ 698.56167, found 698.64 $[M]^{\cdot+}$ and 721.63 $[M+Na]^+$

Synthesis of Poly[9,9-di{(S)-3,7-dimethyloctyl}fluorene-alt-azobenzene] **((*S,S*)-PFAB)**

An oven-dried round-bottom flask connected with a reflux condenser was charged with monomer **PinB-F-BPin** (1000.7 mg, 1.432 mmol, 1.0 eq.), monomer **Br-AB-Br** (487.0 mg, 1.432 mmol, 1.0 eq.), and sodium carbonate (1.523 g, 14.37 mmol, 10.0 eq.). The RBF was evacuated and back-filled with argon three times. $Pd(PPh_3)_4$ (93.7 mg, 0.08 mmol, 0.06 eq.) was added to the RBF under positive argon counter-flow. Then degassed (3 cycles of freeze-pump-thaw) solvent mixture (1,4-dioxane/water, 2 : 1 (*v*/*v*), 37 mL) was added to the RBF and refluxed at 95 °C in the dark under argon for 3 days. After 3 days, a precipitate was formed. **H-F-BPin** (181.5 mg) dissolved in degassed 1,4-dioxane (3 mL, 3 cycles of freeze-pump-thaw)

was added to the RBF and the reaction mixture was stirred at 95 °C for another 18 h. The reaction mixture was cooled down to room temperature and transferred to a separating funnel using chloroform. The organic layer was sequentially extracted with a 1 M HCl (2 × 100 mL), saturated NaHCO3 (2 × 150 mL), deionized water (150 mL), and finally with brine (50 mL). The organic layer was dried over anhydrous sodium sulfate and concentrated in vacuo to give an orange solid. The obtained solid was dissolved in dry THF (10 mL) and diethyldithiocarbamic acid diethylammonium salt as a scavenger agent for residual palladium catalyst (26.9 mg, 0.121 mmol, 0.08 eq.) was added. The red viscous solution was stirred at room temperature under argon for 2 h. The solution was concentrated to ~3 mL in vacuo and excess cold methanol was added to the RBF to obtain an orange solid. The precipitate was filtered under suction and dried overnight at 40 °C in vacuo. The orange solid was subjected to Soxhlet extraction first with acetone (200 mL) at 85 °C for 23 h to remove oligomers, and then with chloroform (200 mL) at 90 °C for 21 h. The chloroform solution was concentrated in vacuo to ~5 mL and excess cold methanol was added to obtain an orange precipitate. The precipitate was filtered and dried overnight at 40 °C in vacuo to give the desired polymer as an orange solid (700.4 mg, 1.121 mmol, 78%).

^{1}H NMR (400 MHz, $CDCl_3$): δ 8.14 – 8.03 (m, 4 H), 7.90 – 7.78 (m, 6 H), 7.73 – 7.63 (m, 4 H), 2.30 – 1.90 (m, 4 H), 1.46 – 1.41 (m, 2H), 1.40 (s, end groups), 1.25 – 1.16 (m, 2 H), 1.15 – 0.85 (m, 12 H), 0.78 (d, J = 6.5 Hz, 12 H), 0.74 (d, J = 6.3 Hz, 6 H), 0.72 – 0.43 (m, 4 H) ppm.

^{13}C NMR (100 MHz, $CDCl_3$): δ 151.9, 144.2, 140.7, 139.3, 132.3, 127.8, 126.3, 124.4, 123.5, 121.5, 120.3, 83.7 (end groups), 55.3, 39.2, 37.6, 36.6, 32.9, 30.6, 27.9, 24.9, 24.7, 22.7, 22.6, 19.6 ppm.

IR (cm^{-1}): ν = 3032 (w), 2951 (m), 2923 (m), 2865 (m), 1596 (m), 1463 (s), 1364 (m), 1226 (m), 1157 (m), 1010 (m), 854 (s), 817 (s)

Elem. Anal. Calcd. for $(C_{45}H_{56}N_2)_n$: C, 86.5; H, 9.0; N, 4.5. Found C, 84.5; H, 8.5; N 4.3.

App. M_n = 12.1 kg/mol, App. M_w = 22.9 kg/mol, $Đ$ = 1.89 (Anal. SEC)

Synthesis of Poly[9,9-di{(R)-3,7-dimethyloctyl}fluorene-alt-azobenzene] **((*R,R*)-PFAB)**: **5** (552 mg, 0.79 mmol, 1.0 eq.), **Br-AB-Br** (268.6 mg, 0.79 mmol, 1.0 eq.) and sodium carbonate (877 mg, 8.3 mmol, 10.5 eq.) were taken in a 3-necked 100 mL RBF fitted with a reflux condenser and an argon inlet. The RBF was evacuated and back-filled with argon (three times). $Pd(PPh_3)_4$ (45 mg, 0.039 mmol, 0.05 eq.) weighed under a positive flow of nitrogen gas was added to the RBF under counter-flow of argon. The RBF was again evacuated and back-filled

with argon (three times). Degassed dioxane:water (30 mL, 2:1, *v*/*v*) solvent mixture (3 cycles of freeze-pump-thaw) was added to the RBF and the flask was heated at 95 °C for 3 days. Then the RBF was allowed to cool down to room temperature and transferred to a separating funnel using chloroform. The organic layer was washed with 1M HCl (3 × 100 mL), saturated $NaHCO_3$ (3 × 100 mL), and finally with deionized water till the aqueous layer was neutral to pH. The collected organic layer was dried over sodium sulfate and concentrated under reduced pressure to obtain an orange solid. To this, diethyldithiocarbamic acid diethylammonium salt (45 mg) and dry THF (25 mL) was added and stirred at room temperature for 15 hours. Then the solution was concentrated to ~5 mL and excess cold methanol was added to the RBF to obtain an orange precipitate. The solids were filtered under suction and dried at 40 °C for few hours. Then the solids were subjected to Soxhlet extraction first with acetone (200 mL at 85 °C) to remove small oligomer and then with chloroform (200 mL at 85 °C) to collect the high molecular weight fractions. The chloroform evaporated under reduced pressure to ~5 mL and excess cold methanol was added to the RBF to precipitate an orange solid. The solids were thoroughly washed with methanol, filtered under suction and dried in a vacuum oven at 60 °C for 15 hours to obtain the desired polymer as an orange powder (345 mg, 67%).

^{1}H NMR (400 MHz, $CDCl_3$): δ 8.08 (m, 4H), 7.85 (m, 6H), 7.71 – 7.67 (m, 4H), 2.12 (m, 4H), 1.46 – 1.34 (m, 3H), 1.2 – 0.9 (m, 15H), 0.78 (d, *J* = 6.28 Hz, 12H), 0.74 (d, *J* = 6 Hz, 6H), 0.71 – 0.44 (m, 4H) ppm.

^{13}C NMR (100 MHz, $CDCl_3$): δ 152.01, 144.33, 140.83, 139.43, 132.50, 127.96, 126.47, 124.53, 123.63, 123.03, 121.63, 120.44, 83.86, 55.42, 39.35, 37.76, 36.76, 33.03, 30.71, 28.07, 25.09, 24.79, 24.72, 22.79, 22.70, 19.74 ppm.

IR (cm^{-1}): ν = 2951 (m), 2923 (m), 2865 (m), 1598 (m), 1464 (s), 854 (s), 817 (s)

App. M_n = 10.1 kg/mol, App. M_w = 17.6 kg/mol, $Đ$ = 1.74 (Anal. SEC)

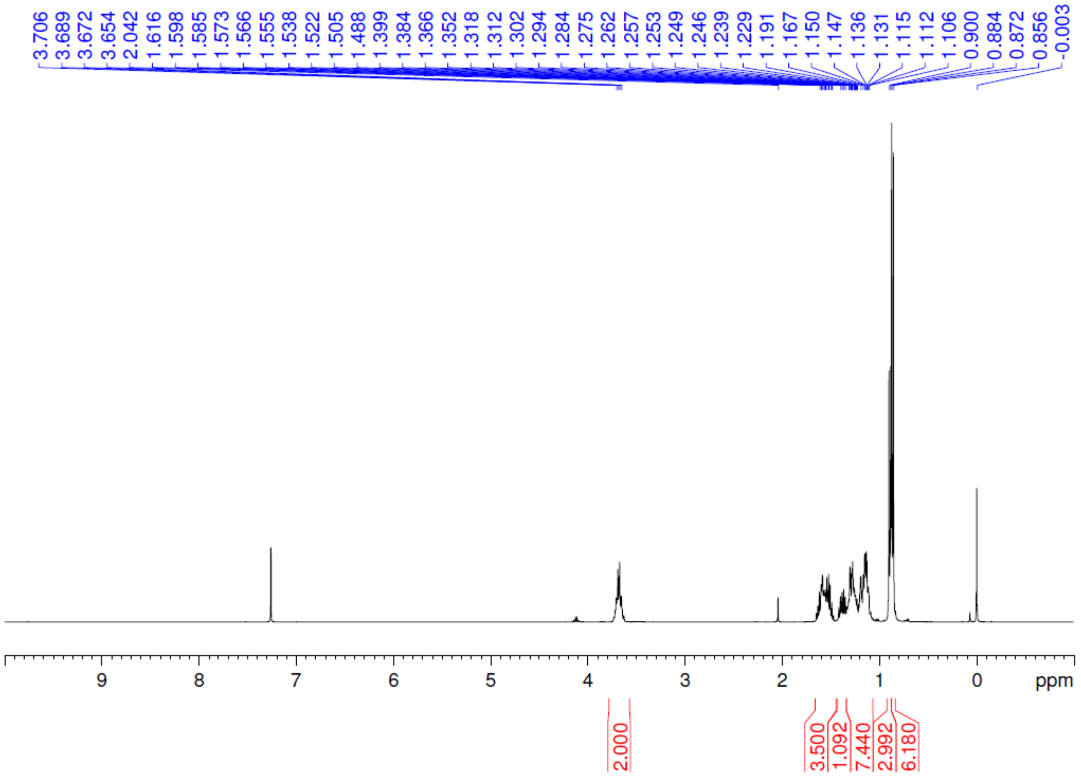

Figure S1: ^{1}H-NMR spectrum (400 MHz, $CDCl_3$) of **2**. The signals after 4 ppm corresponds to residual ethyl acetate solvent.

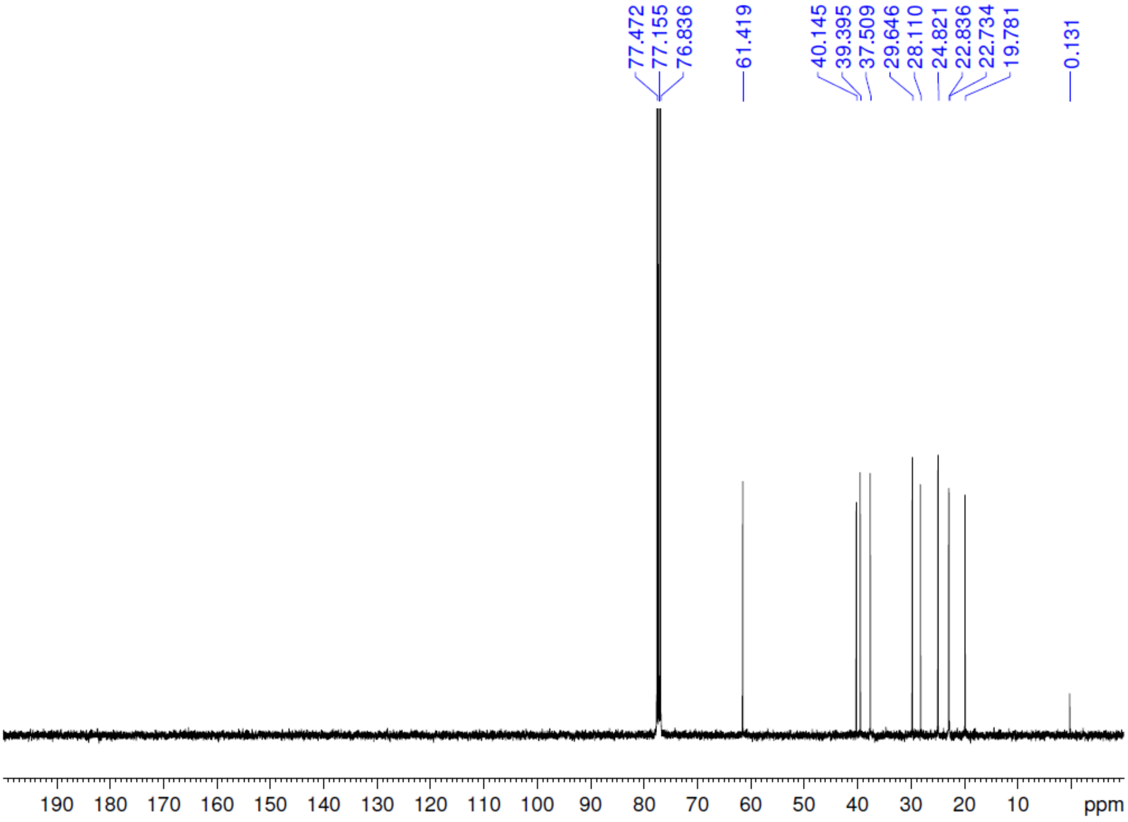

Figure S2: ^{13}C-NMR spectrum (100 MHz, $CDCl_3$) of **2**.

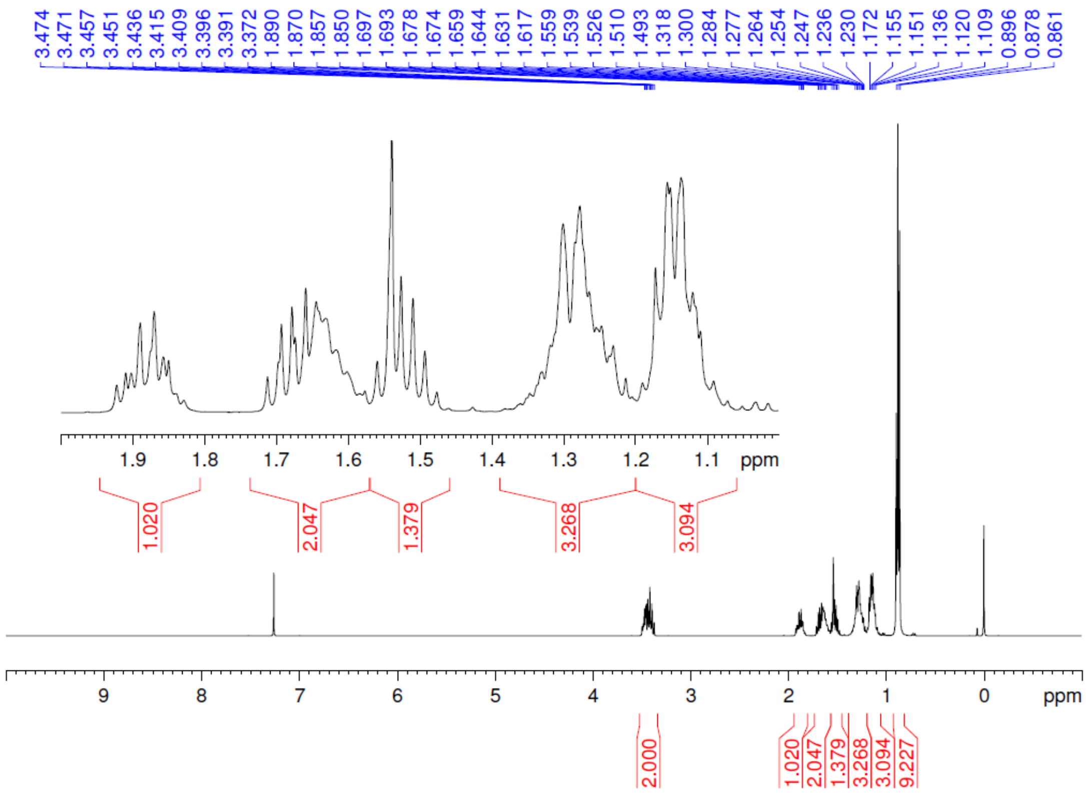

Figure S3: ^{1}H-NMR spectrum (400 MHz, $CDCl_3$) of **3**.

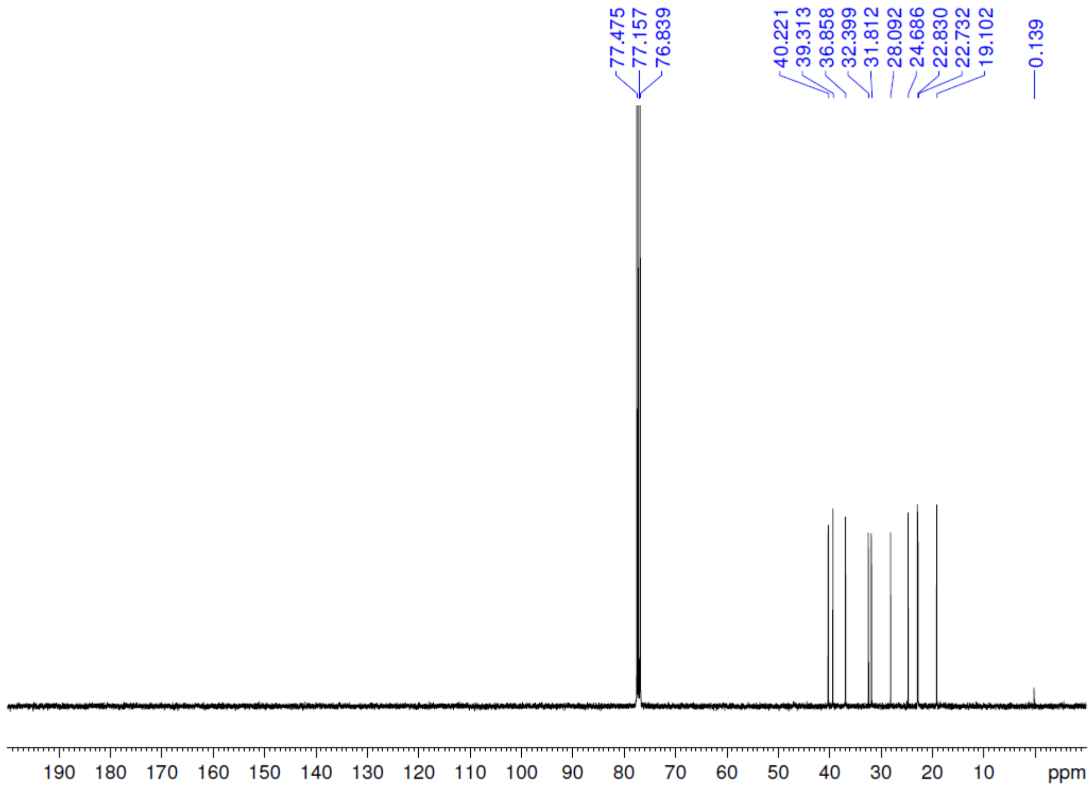

Figure S4: ^{13}C-NMR spectrum (100 MHz, $CDCl_3$) of **3**.

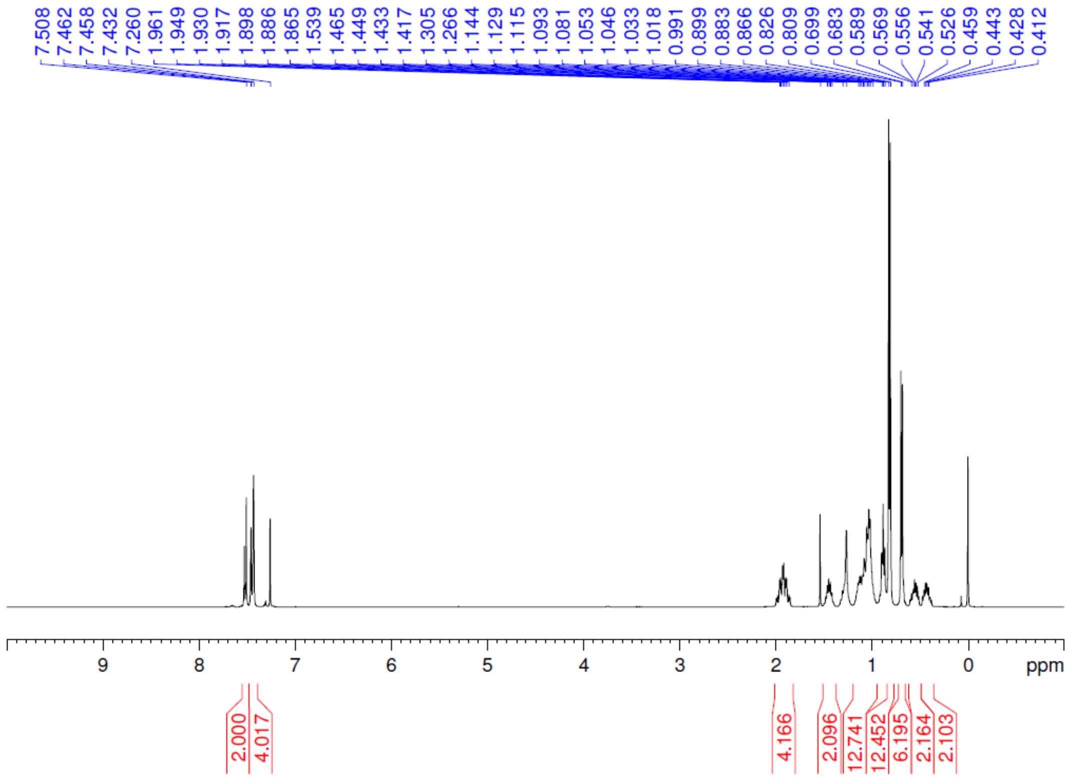

Figure S5: ^{1}H-NMR spectrum (400 MHz, $CDCl_3$) of **4**. There is some amount of residual *n*-heptane accounting for the peaks around 1.25 and 0.88 ppm.

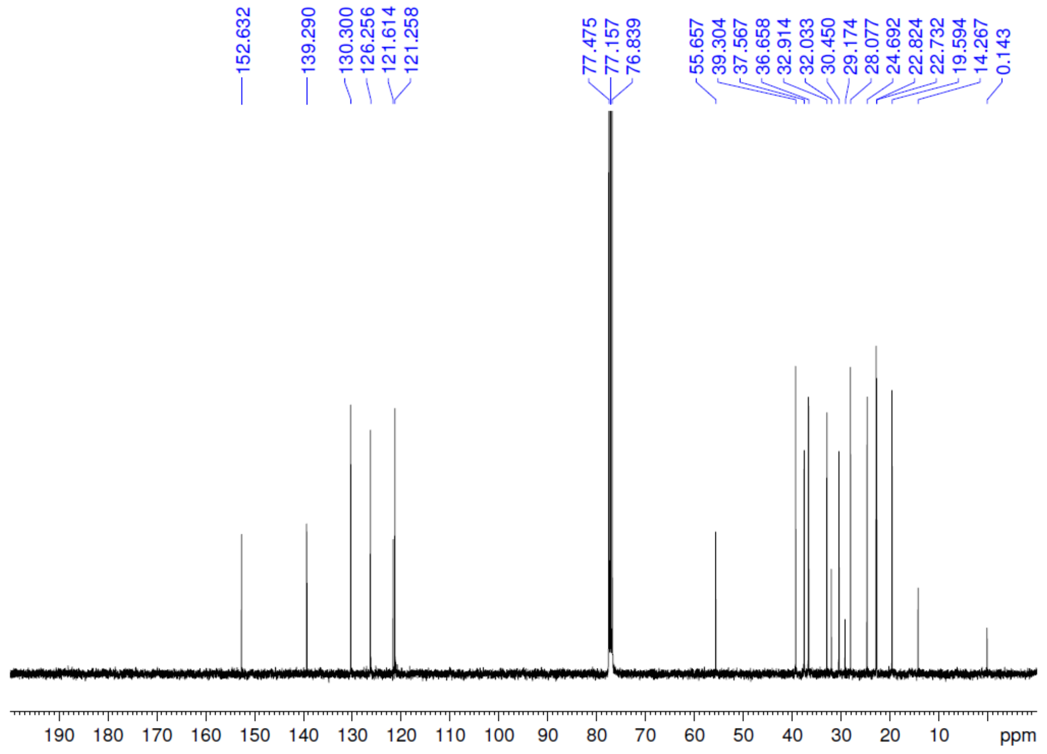

Figure S6: ^{13}C-NMR spectrum (100 MHz, $CDCl_3$) of **4**. The peaks at 32.03, 29.17 and 14.26 ppm are attributed to residual *n*-heptane.

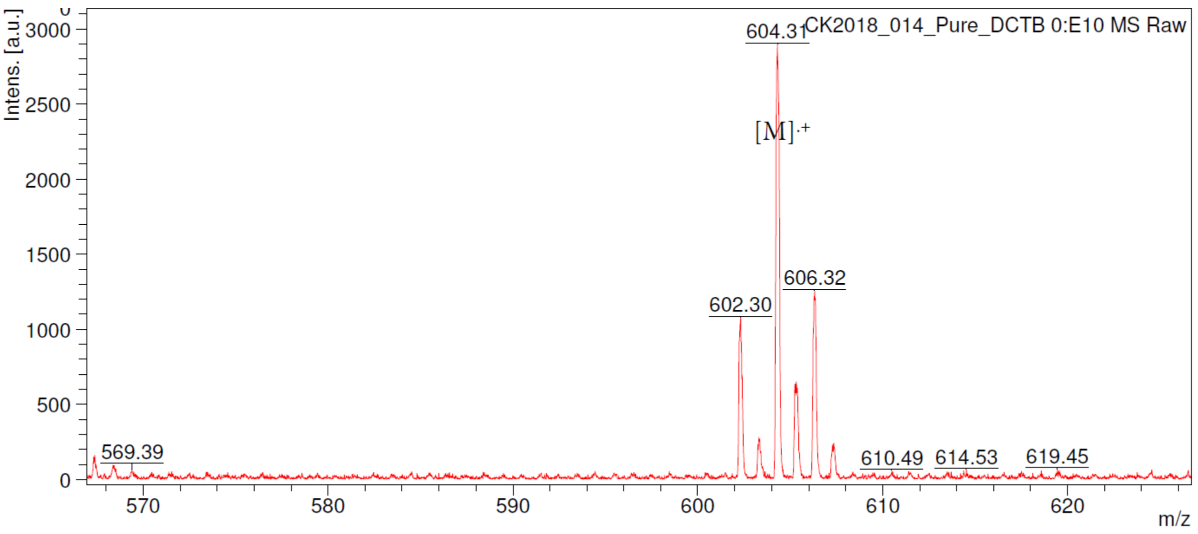

Figure S7: MALDI-TOF Mass spectrum of **4** with DCTB matrix.

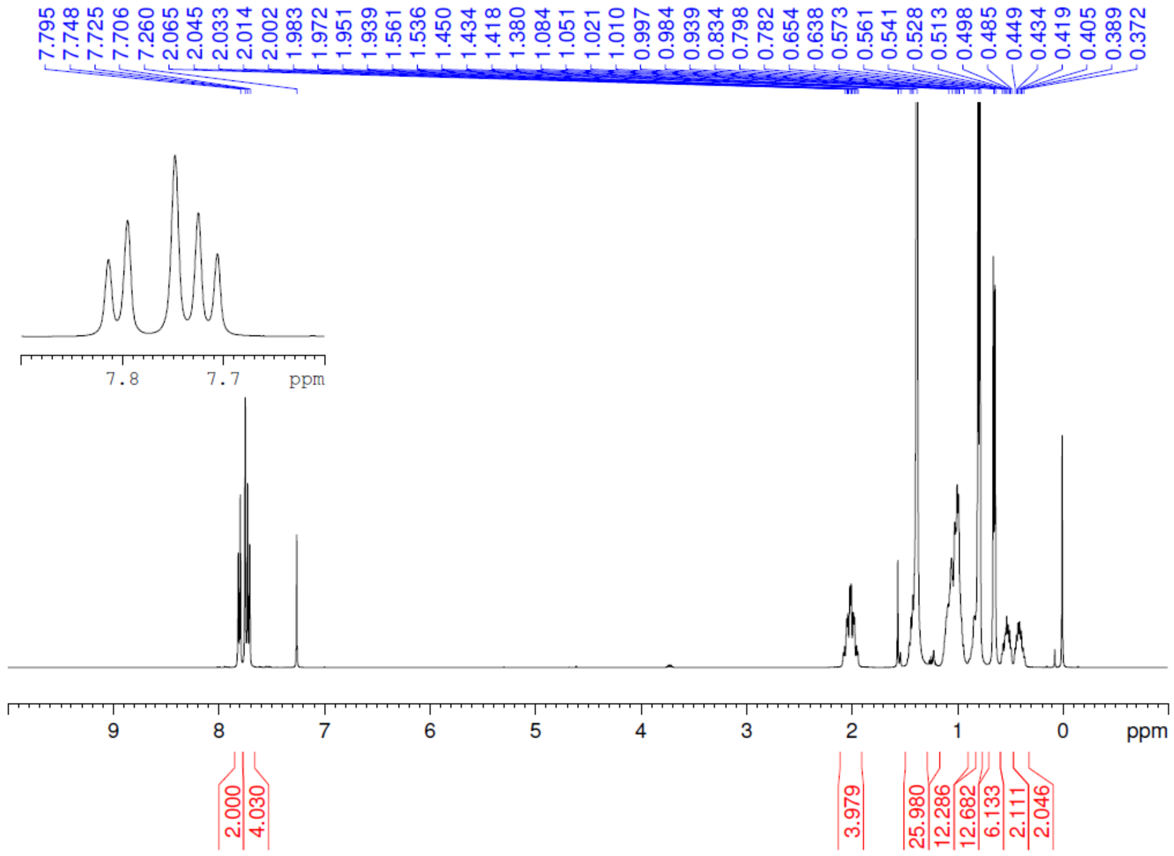

Figure S8: ^{1}H-NMR spectrum (400 MHz, $CDCl_3$) of **5**.

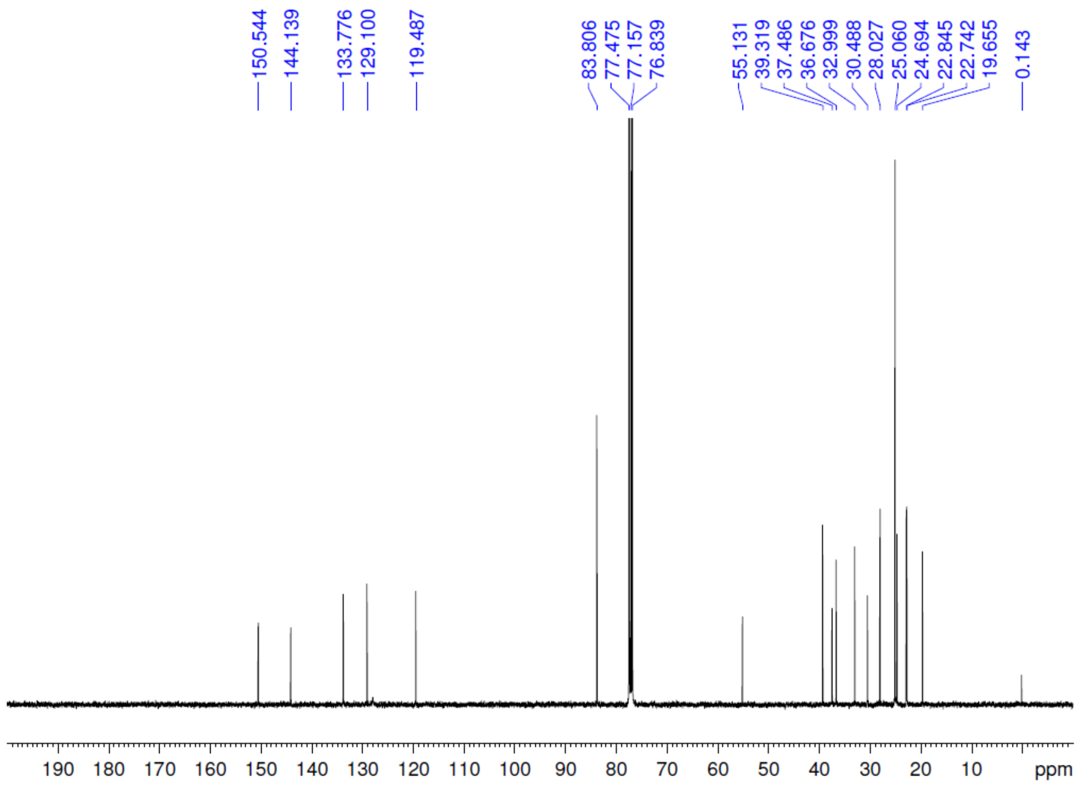

Figure S9: ^{13}C-NMR spectrum (100 MHz, $CDCl_3$) of **5**.

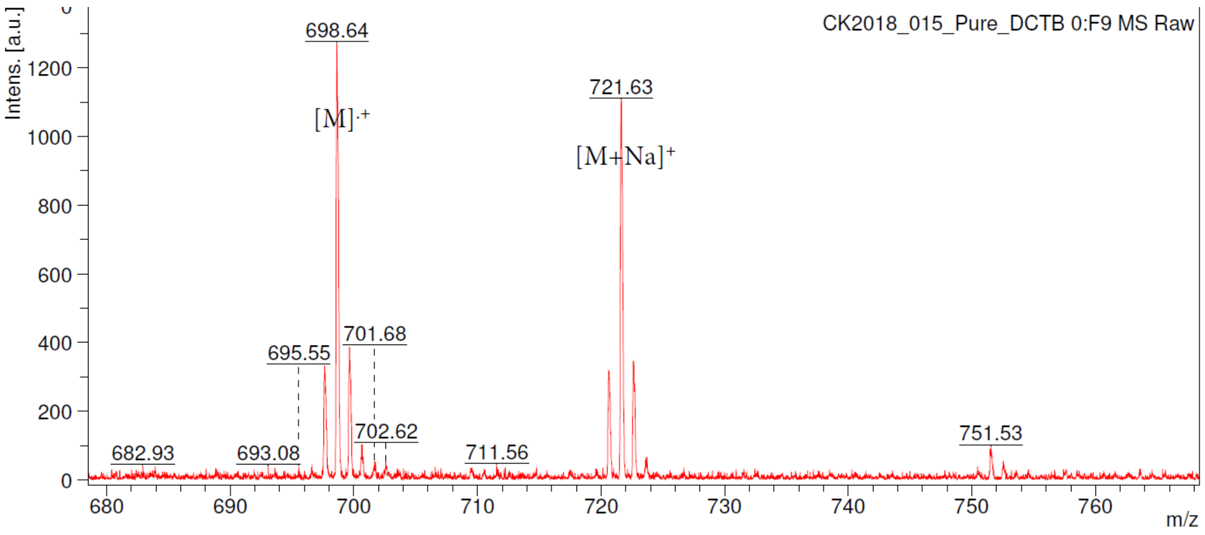

Figure S10: MALDI-TOF mass spectrum of **5** with DCTB matrix.

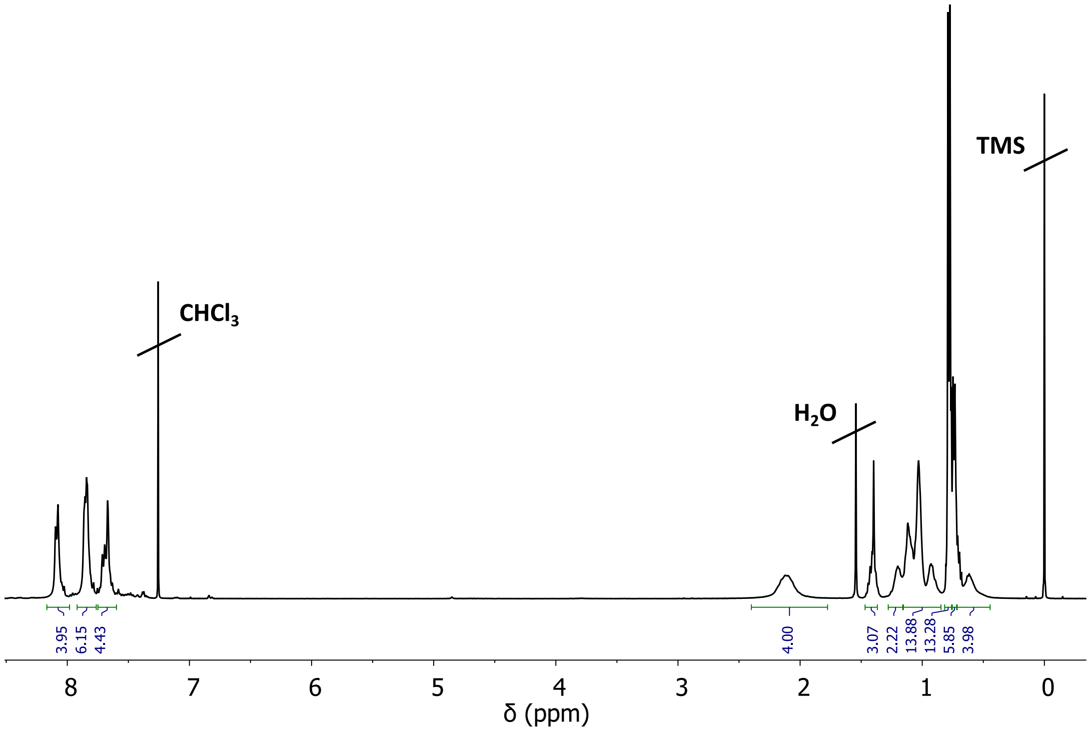

Figure S11. ^{1} H NMR spectrum (400 MHz, CDCl$_3$) of ***(S,S)*-PFAB**.

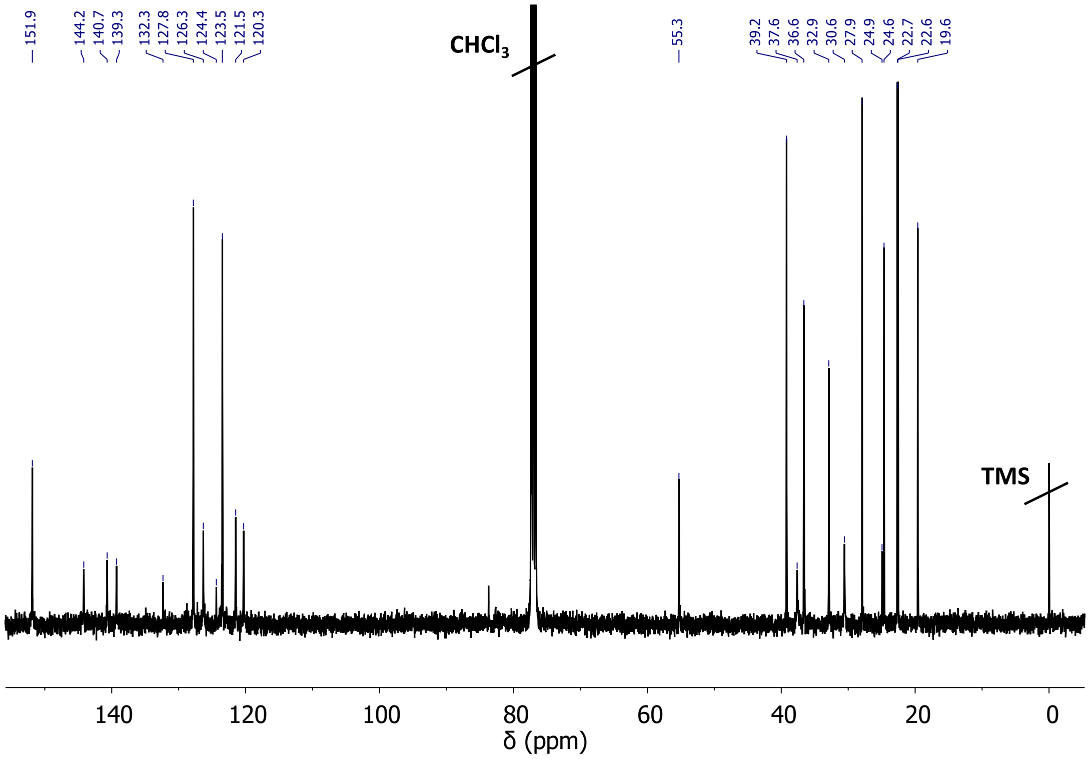

Figure S12. ^{13}C NMR spectrum (100 MHz, CDCl$_3$) of ***(S,S)*-PFAB**.

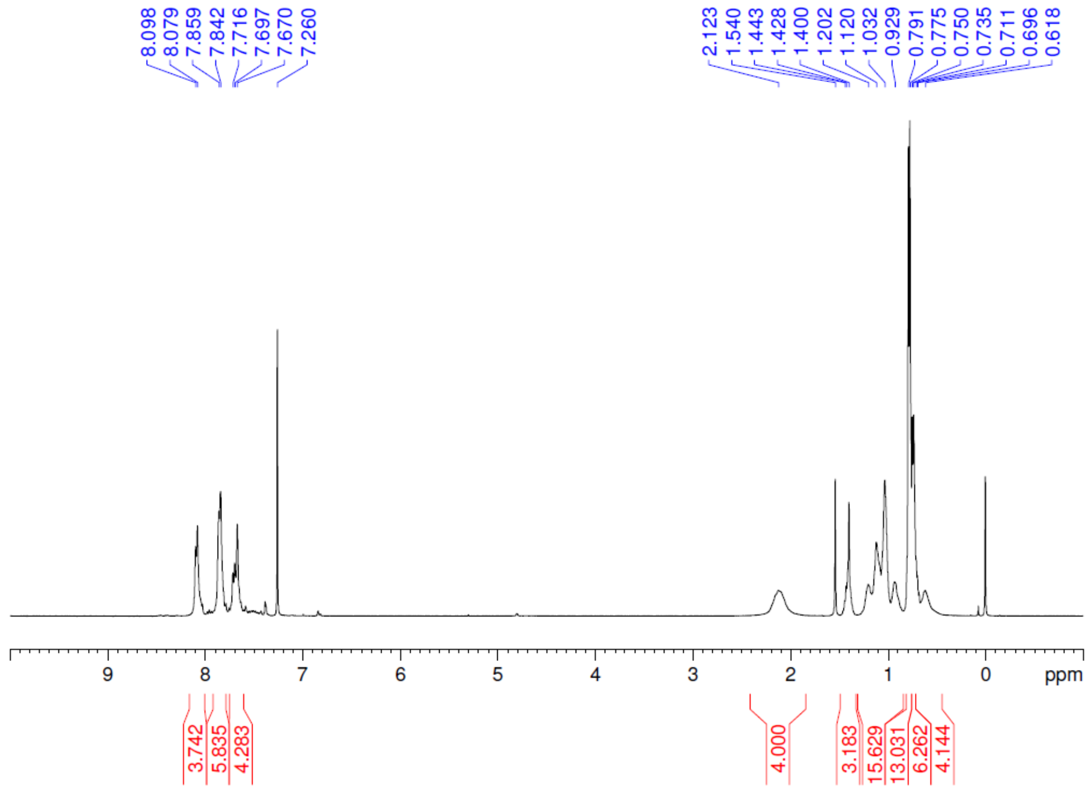

Figure S13: ^{1}H-NMR spectrum (400 MHz, $CDCl_3$) of ***(R,R)*-PFAB**.

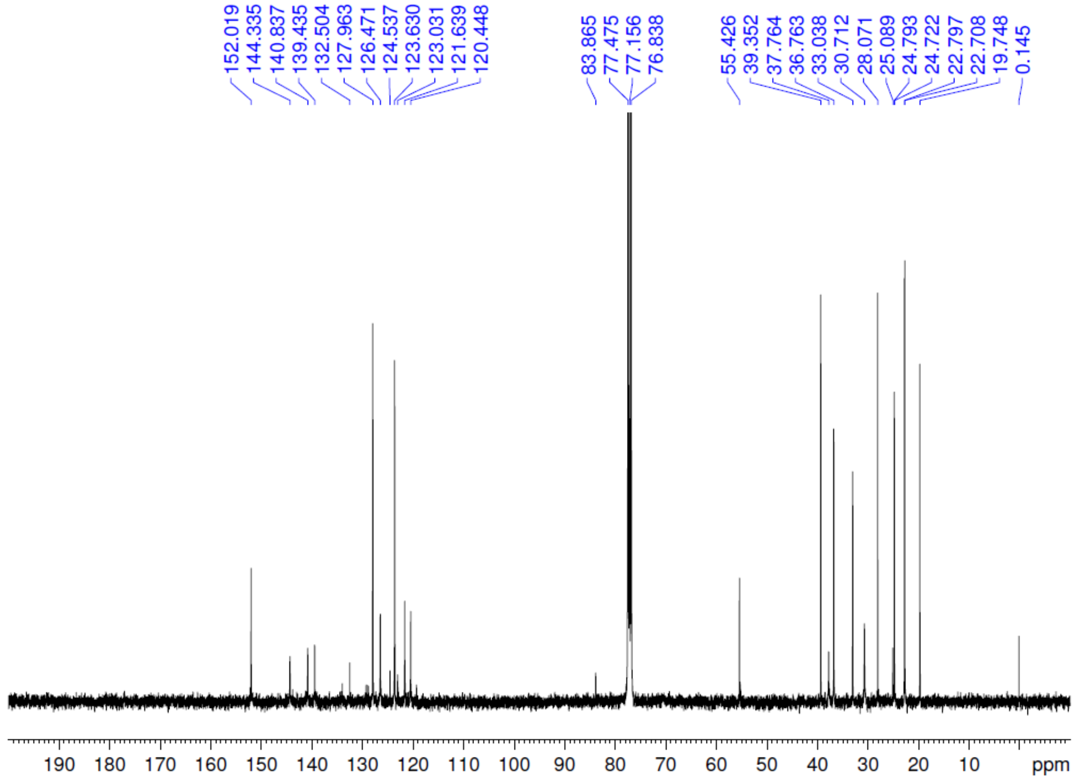

Figure S14: ^{13}C-NMR spectrum (100 MHz, $CDCl_3$) of ***(R,R)*-PFAB**.

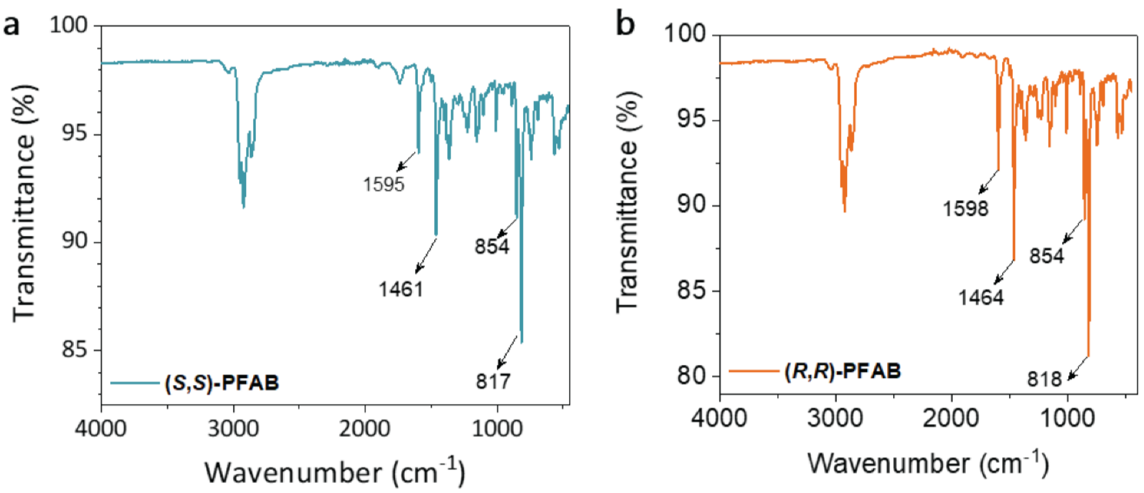

Figure S15: a) and b) Solid-state FT-IR spectrum of ***(S,S)*-PFAB** and ***(R,R)*-PFAB**, respectively.

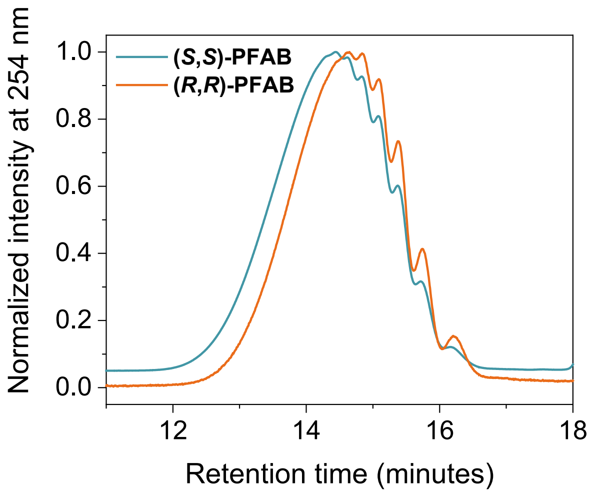

Figure S16: Size exclusion chromatogram (SEC) trace of ***(S,S)*-** and ***(R,R)*-PFAB** using polystyrene as the standard in THF. It can be observed that both enantiomeric forms of polymer have similar molecular weight distributions.

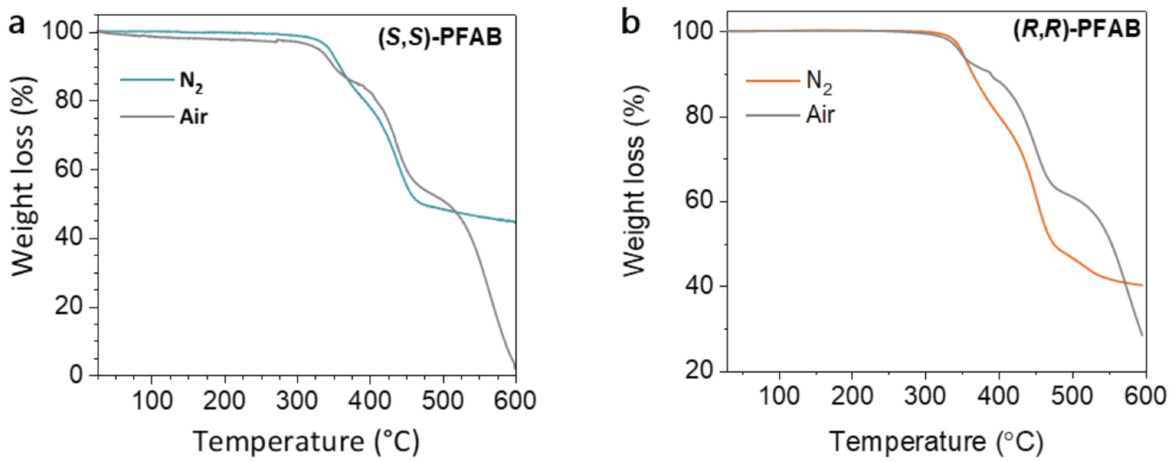

Figure S17: a) and b) Thermogravimetric analysis of **(*S*,*S*)-PFAB** and **(*R*,*R*)-PFAB**, respectively under different conditions. A heating rate of 10 °C/min was employed.

We observe that up to 300 °C the weight loss is < 5%, indicating a high thermal stability of the polymer.

3. Supplementary Figures

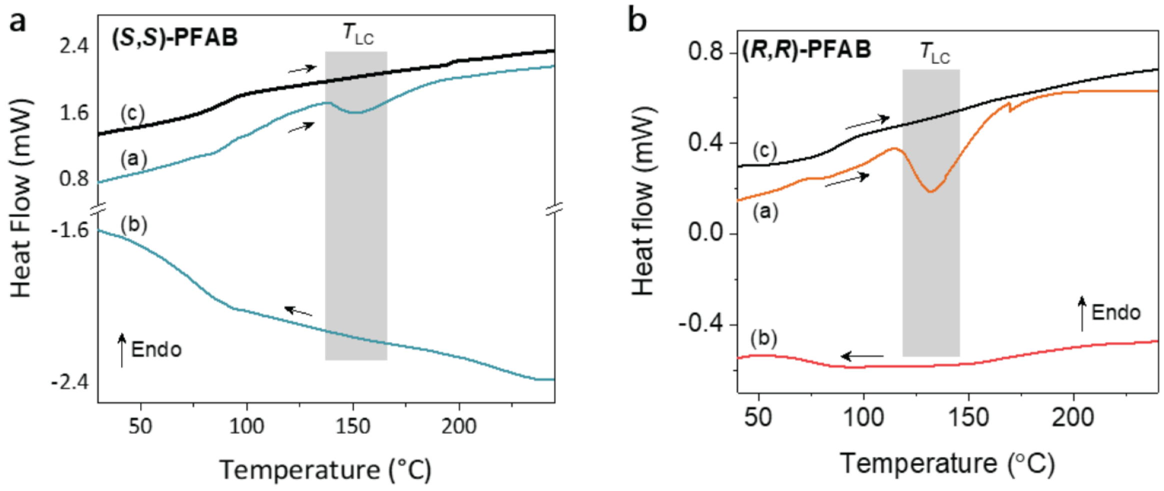

Figure S18: a) and b) DSC profile of **(*S,S*)-PFAB** and **(*R,R*)-PFAB**, respectively. (a) First heating run, (b) First cooling run and, (c) Second heating run. All the runs were recorded at 10 °C/min. The region of the most prominent transition are depicted in the gray bar.

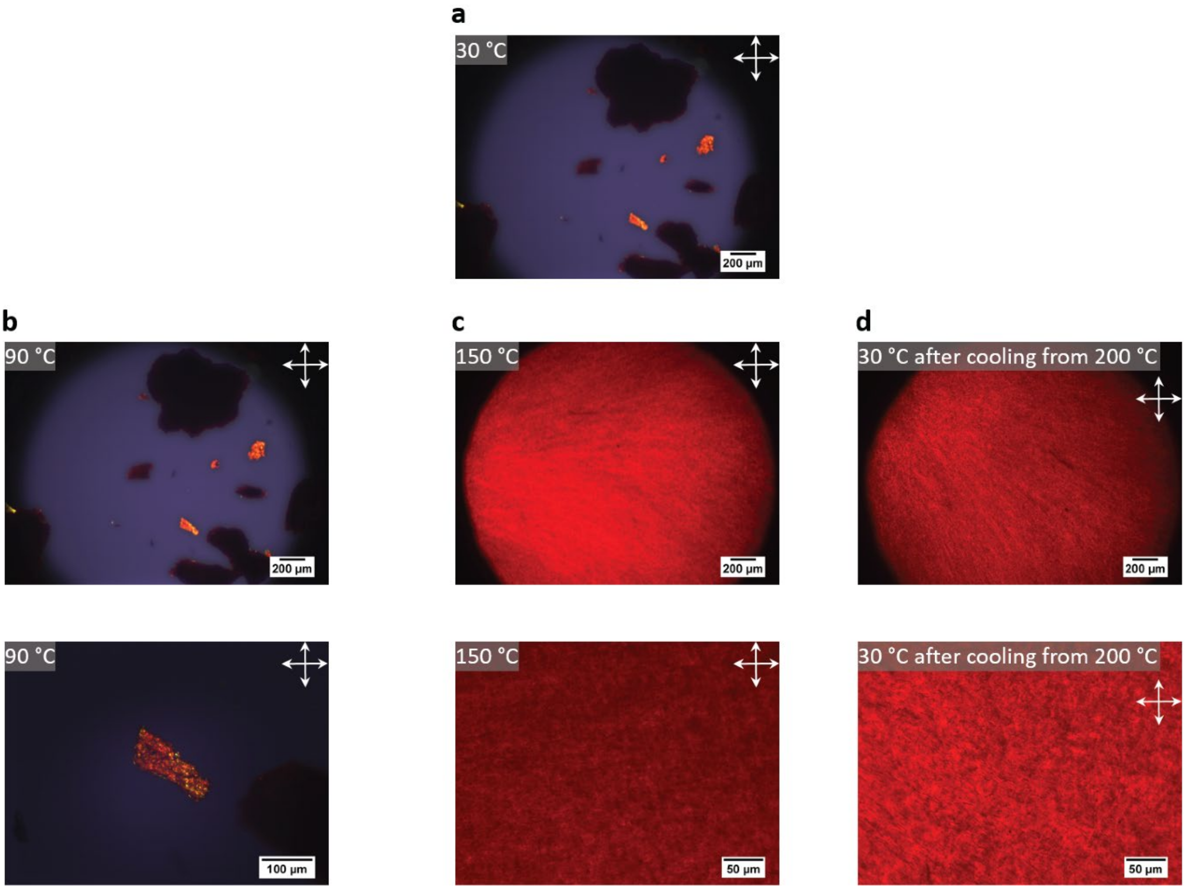

Figure S19: Bulk polarized optical microscopy (POM) images of ***(S,S)*-PFAB**. A small amount of as synthesized ***(S,S)*-PFAB** was placed between two glass cover slips and viewed between cross-polarizers. (a) POM image at room temperature before heating. (b) POM image at 90 °C (c) POM image at 150-160 °C, near the transition observed in the DSC. (d) POM image at 30 °C, after cooling from 200 °C. For (b), (c) and, (d) the top and bottom panels represent POM images at different magnification.

It can be observed that some of the grains of the as synthesized polymer are birefringent. On heating at 150-160 °C resulted in increased birefringence and also the viscosity of the polymer decreased on shearing. On further heating beyond 160 °C, no significant changes in birefringence or physical form of the polymer was observed until 200 °C. On cooling the sample from 200 °C to room temperature, the birefringence was retained, and it did not change on further heating. This is consistent with the transition around 150 °C which is only observed in the first heating cycle of DSC (Figure S19). Thus we conclude that around 150 °C, ***(S,S)*-PFAB** undergoes a transition into a liquid crystalline state, which is retained upon cooling. Similar observations have been made for other fluorene-based polymers.

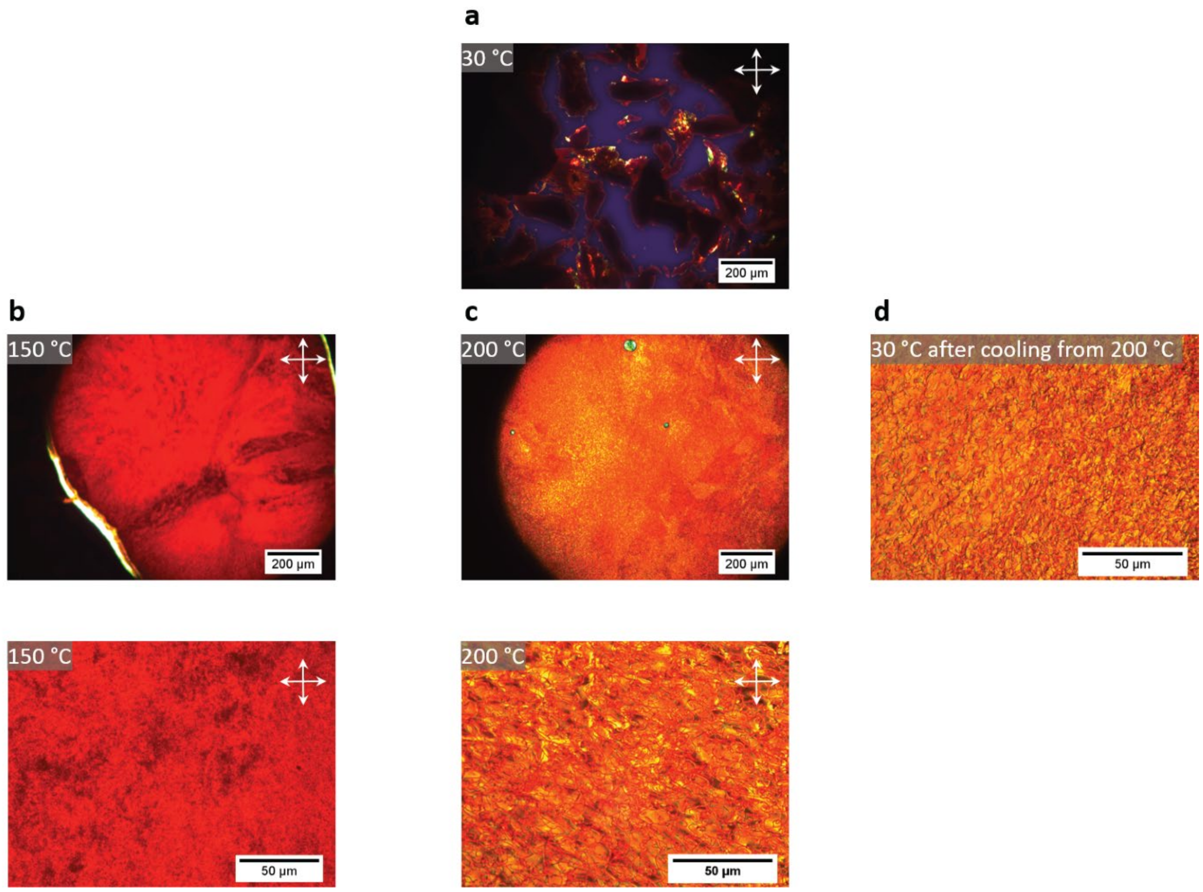

Figure S20: Bulk polarized optical microscopy (POM) images of ***(R,R)*-PFAB**. A small amount of as synthesized ***(R,R)*-PFAB** was placed between two glass cover slips and viewed between cross-polarizers. (a) POM image at room temperature before heating. (b) POM image at 150 °C, near the transition observed in the DSC (c) POM image at 200 °C (d) POM image at 30 °C, after the cooling from 200 °C. For (b) and (c) the top and bottom panels represent POM images at different magnification.

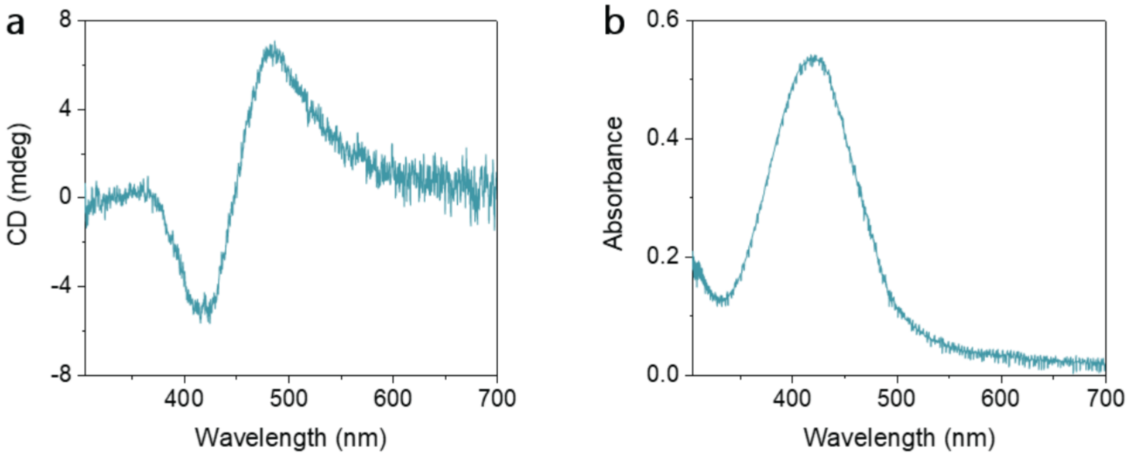

Figure S21: a) and b) CD and UV-vis spectra, respectively of unannealed **(*S*,*S*)-PFAB** film. Typical film thickness is ~60 nm.

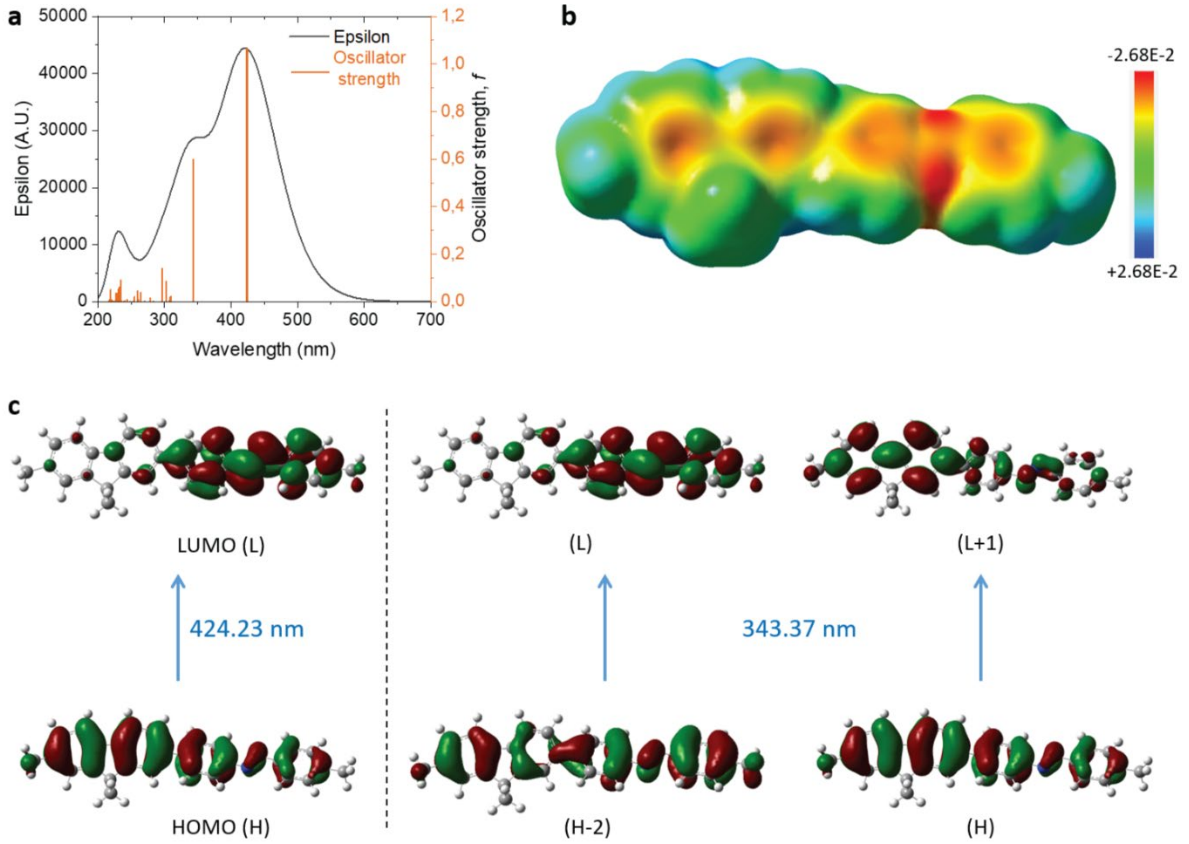

Figure S22: Theoretical computation on model compound of *trans*-conformer of **(*S,S*)-PFAB** repeat unit carried out in gas phase at zero Kelvin. The side chains were replaced by methyl groups to reduce the computations costs. a) Computed UV-vis spectra of the model compound using time-dependent DFT (36 states). Both the epsilon and oscillator are shown in the graph. The FWHM is chosen to be 0.333 eV = 2688 cm^{-1}. b) Electrostatic potential surfaces on a constant density surface using Merz-Kollmann charges. c) Frontier molecular orbitals of the two highest oscillator strength transitions. All the computations were carried out at B3LYP/6-31+G(d,p) level of theory.

It can be clearly observed that the transition with the highest oscillator strength (~425 nm) matches very well with the experimental UV-vis maximum (~420 nm) observed in thin film. Although the azobenzene group is in conjugation with the fluorene, a significant electron density is available around the azo-group for photoisomerization.

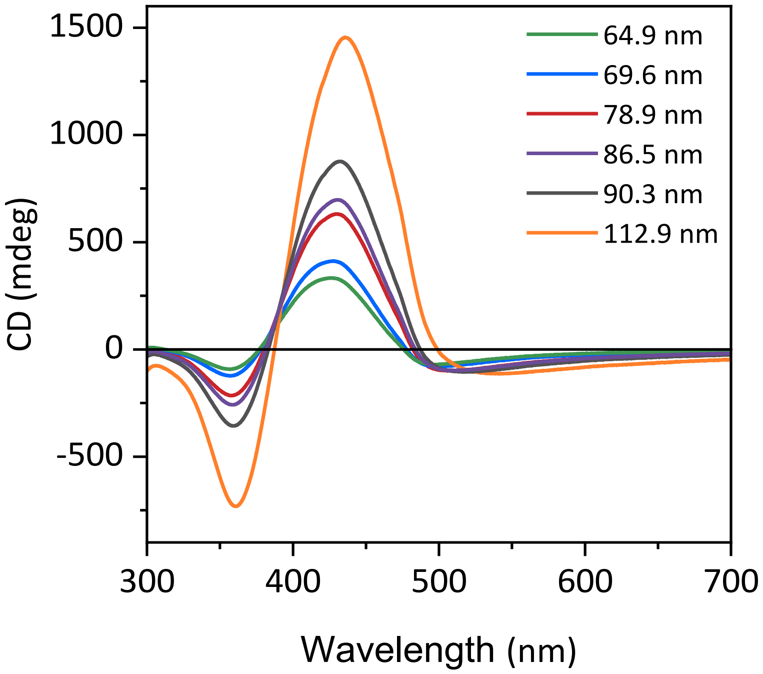

Figure S23: Room temperature CD spectra of **(*S*,*S*)-PFAB** as a function of film-thickness.

It can be observed from the above graph that, the magnitude of CD increases monotonically as the film thickness is increased. For thickness above ~120 nm, the CD exceeds 2000 mdeg and it cannot be measured by the commercial CD spectrophotometers (J-815). Thus, for all film thickness above 120 nm, the g_{abs} was calculated using spectroscopic ellipsometry by a previously reported procedure.[7]

Probing the supramolecular organization by LD spectroscopy

In accordance with our previous work,[8] polarized transmission and reflection measurements were performed. First, thin film of different polymer layer thickness were prepared by spin-coating the polymer on clean glass slides and planar rubbed polyimide glass slides. A similar thickness of the polymer layer was assumed for rubbed polyimide thin film compared to the polymer layer on clean glass slides prepared under the exact same conditions. All measurements were performed on the rubbed polyimide thin film with the polymer layer facing the light source. Reflection measurements were conducted with a 15° angle of incidence. It was assumed that the direction of polarization of light in transmission was unaffected by passing through the thin film. Two orientations were chosen: the rubbing direction parallel to the vertical axis (*rub V*) and the rubbing direction rotated over +45° w.r.t. the vertical axis as seen from the light source (*rub cw 45°*).

First, the handedness of the cholesteric arrangement was determined by analyzing polarized transmission and reflection spectra of a 60 nm aligned thin film of **(*S,S*)-PFAB**. Both film orientations are shown (Figure S24a), in which the left column represents the rubbing direction parallel to the vertical axis and the right column the rubbing direction rotated over 45° clockwise. The minimum in transmission at 422 nm for both polarizations and thin film orientations corresponds to the absorption maximum of **(*S,S*)-PFAB** observed in the absorption spectrum. In case of the vertical orientation of the rubbing direction, the transmission of the vertical polarization of light is significantly lower compared to the horizontal polarization (Figure S24b left panel). This is to be expected, because the polymer chains will be mostly aligned in the vertical direction and thus absorb more vertically polarized light leading to a lower transmission. In addition, the reflection of vertically oriented polymer films is strongly polarized in the vertical direction. Upon rotating the thin film over 45° in the clockwise direction, the transmission in the vertical direction increases, whereas the transmission in the horizontal direction slightly decreases. In reflection, there is a bias for vertical polarized light much like the aforementioned film orientation (Figure S24b, right panel). This indicates that the polymer chains tend to rotate in the counterclockwise direction and thereby prefer the absorption of vertical polarized light, which corresponds to a right-handed cholesteric arrangement of **(*S,S*)-PFAB**.

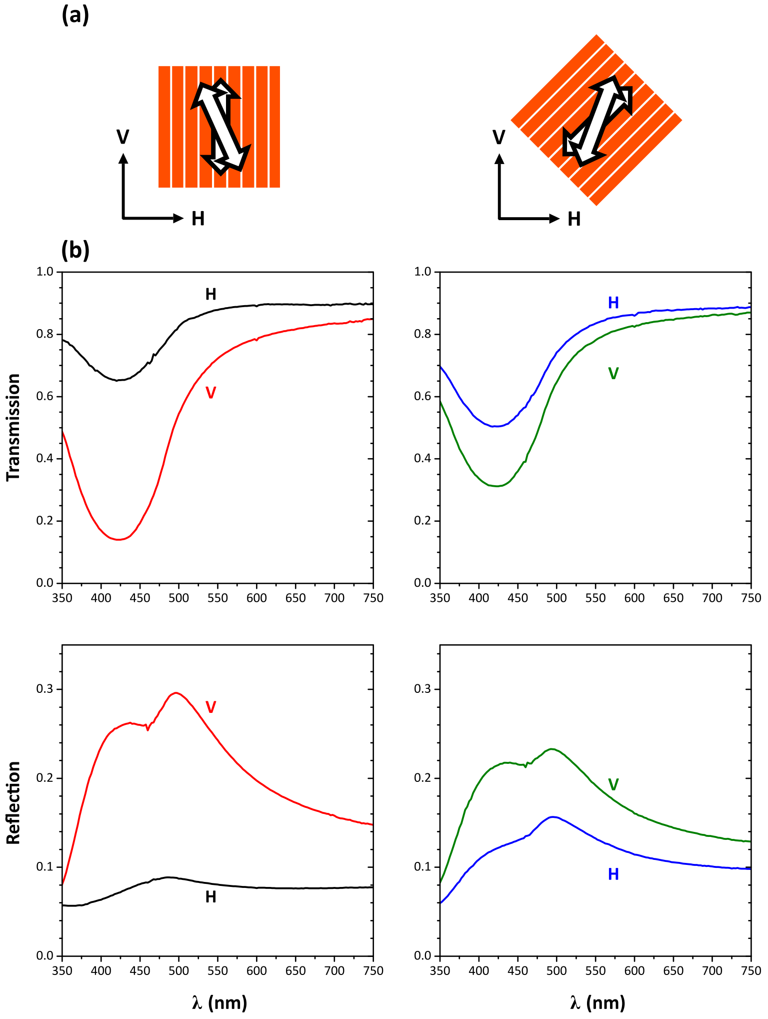

Figure S24: (a) Schematic of the direction of the alignment layer and the orientation of the transition dipole moments of the polymer chains in thin film. (b) Linear dichroism in transmission and reflection for a 60 nm aligned thin film of **(*S,S*)-PFAB** in both orientations as depicted in the schematic. V: vertical polarization, H: horizontal polarization.

Three distinct thicknesses of films were chosen to investigate the pitch of the cholesteric handedness. These three characteristic thicknesses are schematically depicted in Figure S25a as: (I) d = one-eighth of the pitch, (II) d = one-fourth of the pitch, and (III) d = one-half of the pitch. Correspondingly, the transition dipole moment of the polymer chains rotates over 45°, 90°, and 180°, respectively.

The degree of linear polarization in reflection as a function of film thickness for both thin film orientations (*rub V*: black squares, *rub cw 45°*: red circles) is depicted in Figure S25b. The experimental data was fitted to the Good-Karali model[9] to extract the pitch of the cholesterics. As can be seen, the degree of linear polarization in reflection is greatest (0.57) for the lowest

film thickness. By increasing the film thickness, an oscillatory pattern tends to emerge. Fairly good agreement between the data points and the fitting curves can be obtained if a right-handed cholesteric arrangement of **(*S*,*S*)-PFAB** with a helical pitch length of 1600 nm is assumed. However, in some cases significant deviations of the data points w.r.t. the fitting curves are observed. For example, a difference of 0.54 is seen for a 420 nm aligned thin film in the rotated film orientation (*rub cw 45°*). This is related to the fact that only the polymer chains at the top surface are responsible for the reflection. It is highly likely that the polymer chains align in a superior manner near the alignment layer compared to the top surface. Nevertheless, the trend of the fitting curves can be qualitatively understood.

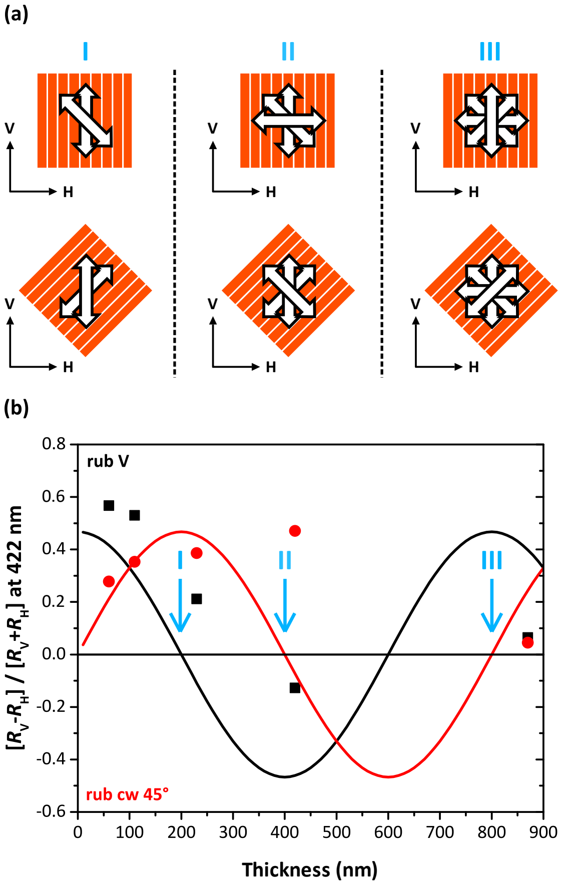

Figure S25. (a) Schematic of the right-handed cholesteric arrangement of polymer chains in both film orientations (Top row: *rub V*, bottom row: *rub cw 45°*) for three different thicknesses. (b) Degree of linear polarization in reflection (15° angle of incidence) of vertically and horizontally polarized light through aligned films of **(*S*,*S*)-PFAB**. Lines show predictions of Eq. 5 from ref. [8] with pitch length = 1600 nm. The maximum degree of circular polarization has been scaled to down to ± 0.47.

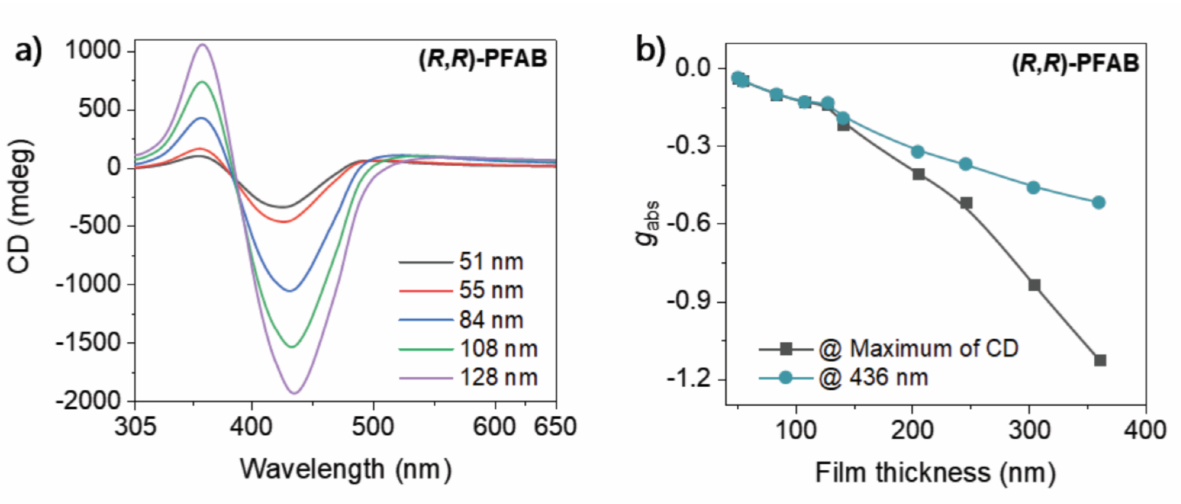

Figure S26: a) and b) Thickness-dependent CD and g_{abs} spectra, respectively of **(*R*,*R*)-PFAB** annealed films. All the samples were annealed at 150 °C for 15 minutes and the spectra are measured at room temperature.

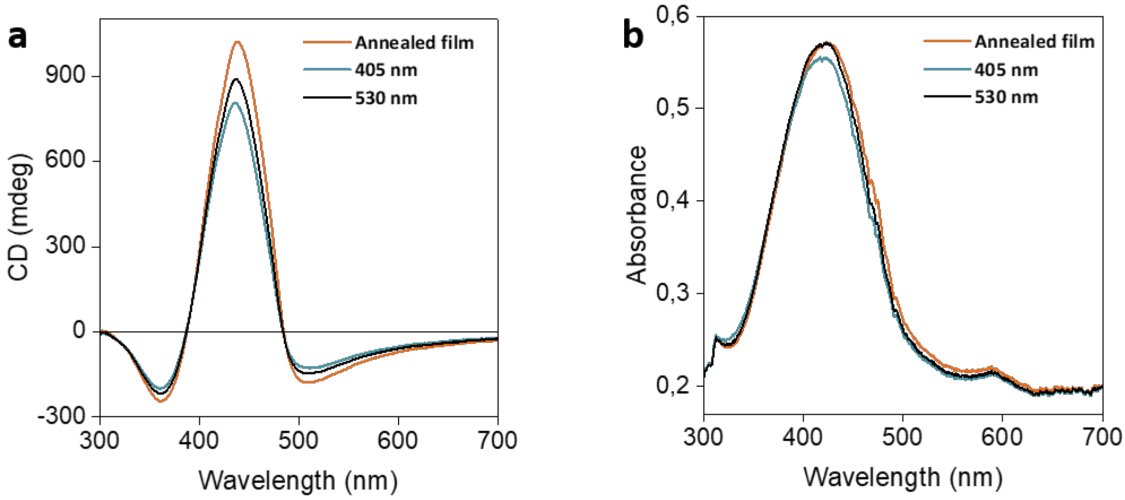

Figure S27: a) and b) CD and UV-Vis spectra, respectively of **(*S,S*)-PFAB** annealed film with or without irradiation. The annealed film was irradiated with 405 and 530 nm LED (27±2 mW/cm^2) without solvent vapor annealing (SVA). Film thickness ~70 nm.

From the above graph, although we see a drop in the CD effect with 405 nm irradiation (blue trace) and partial recovery with 530 nm irradiation (black trace), the magnitude of change in both CD and UV-Vis spectra is very minimal (< 10%). This clearly suggests inefficient photoisomerization of azobenzenes in **(*S,S*)-PFAB**. The area of irradiated part was larger than the beam size of CD spectrometer, thus ruling out that the change in CD and UV-vis are not due to measurement errors. Alternating cycles were performed with both light sources in which CD spectra were recorded at 20 °C after each step.

Experimental set-up of solvent vapor annealing (SVA) process:

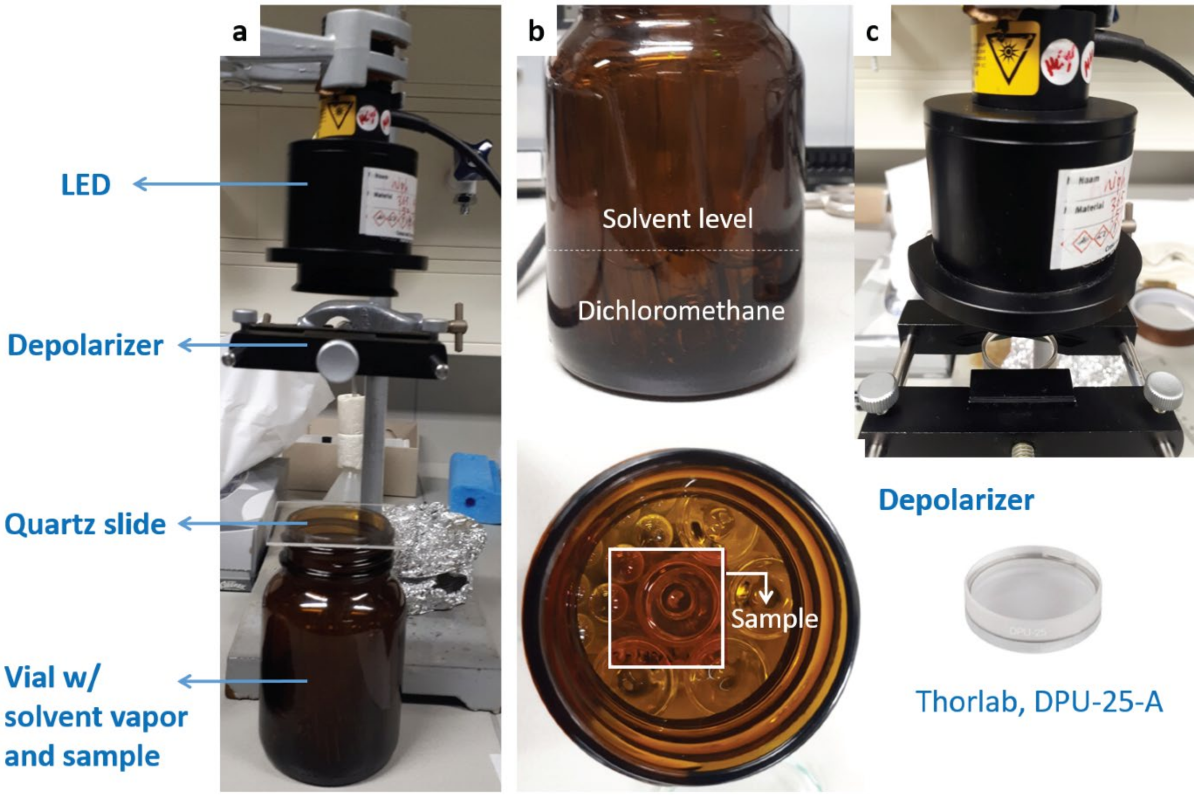

Figure S28: The experimental set-up used for solvent vapor annealing (SVA). a) The full experimental set-up used for irradiation with pseudo unpolarized light. b) Zoom in on the vial. Here an amber colored glass vial was first filled with smaller sample vials (inverted) as a platform to hold the sample. Then dichloromethane was poured into the amber colored vial such that the level of the solvent is well below the base of the smaller sample vials. Then the sample is placed on the base of these smaller sample vials and was covered with a quartz slide. c) For pseudo unpolarized light experiments, the irradiation was carried out with a depolarizer (DPU-25-A from Thorlabs). The depolarizer image was obtained from the website of Thorlabs (https://www.thorlabs.com/thorproduct.cfm?partnumber=DPU-25-A).

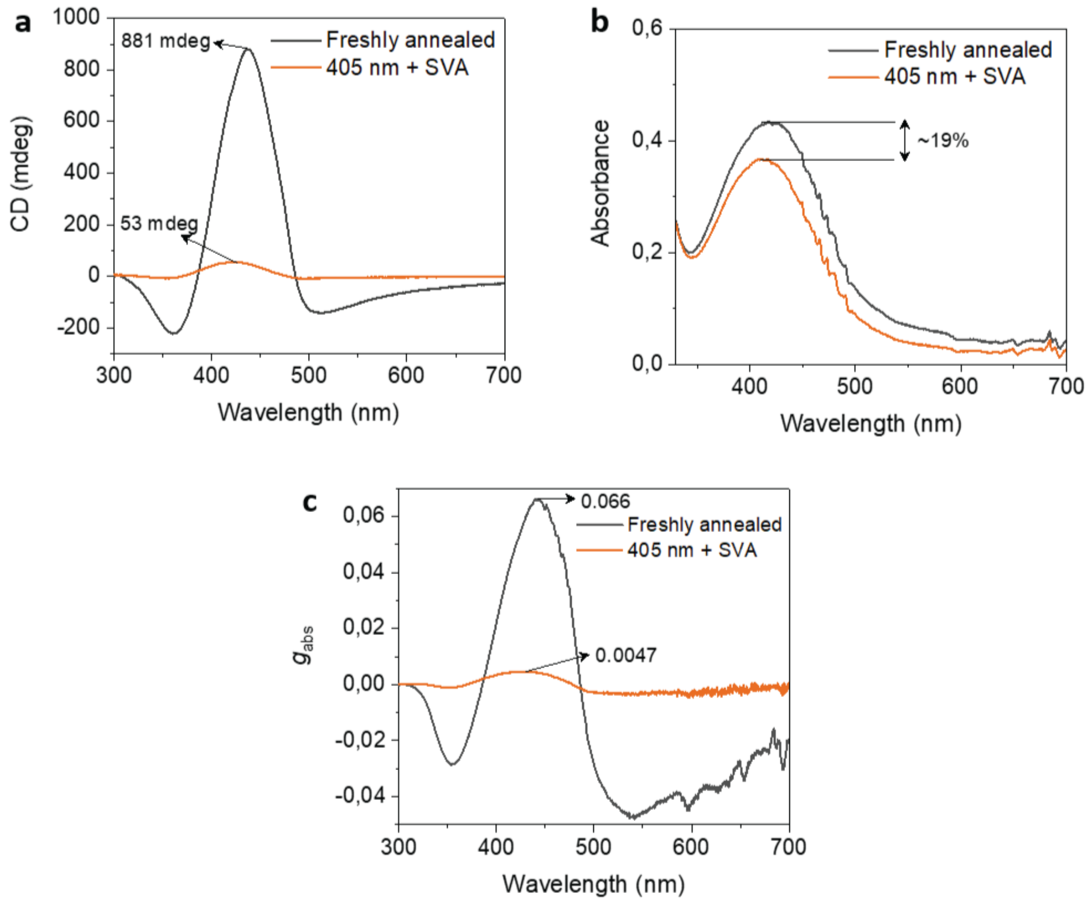

Figure S29: Effect of solvent vapor annealing on chiroptical properties. a), b) and c) CD, UV-vis and gabs spectra, respectively of ***(S,S)*-PFAB** film freshly annealed and 405 nm irradiation with SVA for 15 minutes. All the studies were done without the depolarizer. The intensity of incident light is 27 ± 2 mW/cm^2. Film thickness is 70 nm.

It can be observed from the above figure that irradiation in presence of SVA leads to significant changes in CD and UV-vis spectra. A small (5-10 nm), but definite bathochromophic shift and drop in absorption at the maximum (420 nm) by ~19%, are attributed to the photoisomerization of azobenzenes ***(S,S)*-PFAB**. Increasing the exposure time and intensity did not lead to changes in CD and UV-Vis spectra.

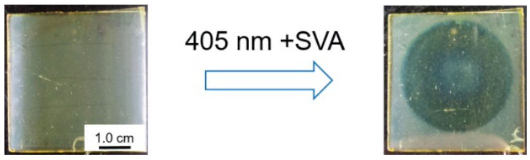

Figure S30: Pictures showing the macroscopic change in the transparency of the film on irradiating with 405 nm + SVA. It can be clearly seen that the irradiated area becomes more transparent. This is reflected in the lower absorbance at higher wavelength in the UV-Vis spectra (Figure S29b).

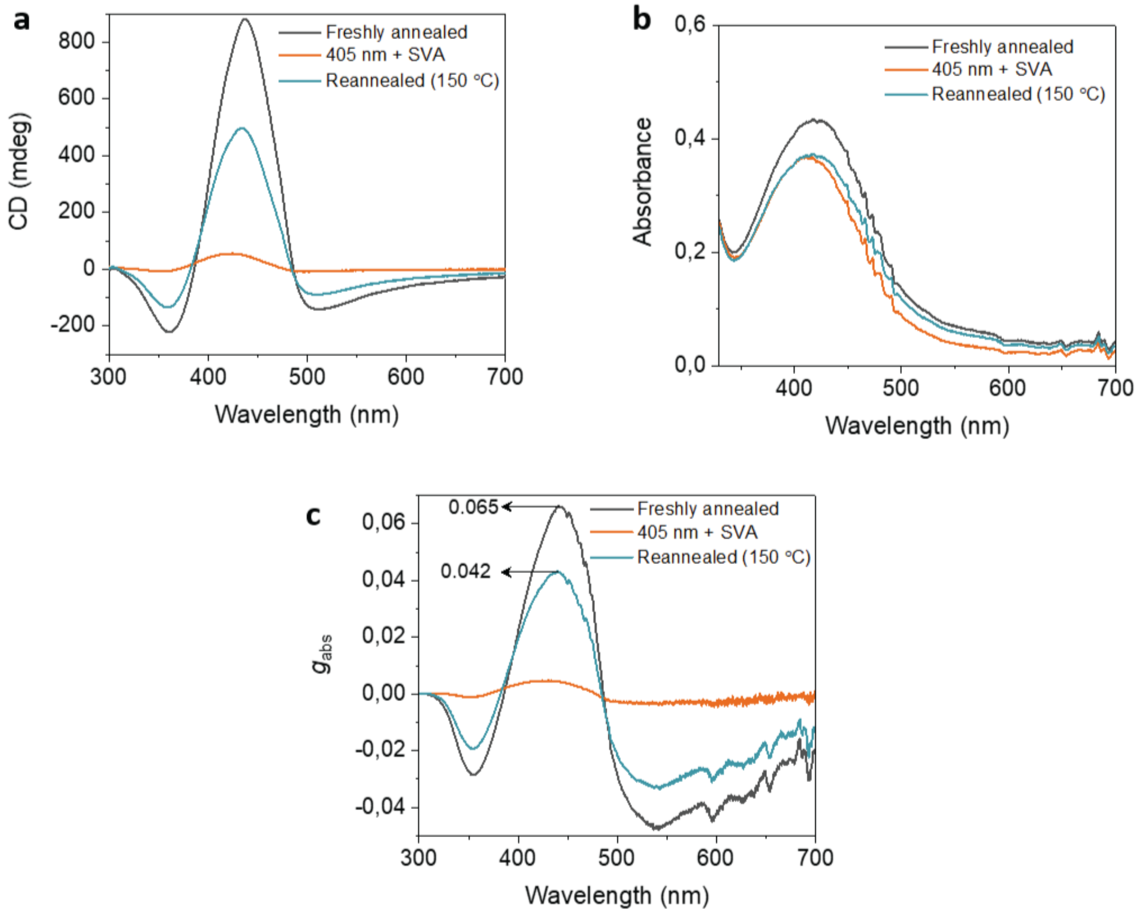

Figure S31: Effect of reannealing on an irradiated **(*S,S*)-PFAB** film. a), b), and c) CD, UV-vis, and g_{abs} spectra of **(*S,S*)-PFAB** on reannealing at 150 °C for 15 minutes. Film thickness is 70 nm.

It can be observed from the above graph that, on annealing an irradiated **(*S,S*)-PFAB** film at 150 °C for 15 minutes we observe that the magnitude of CD effect recovers to roughly half of its pre-irradiated value. The normalized g_{abs} recovers by ~60% (from 0.065 to 0.042). Although the CD effect recovers, it is not complete.

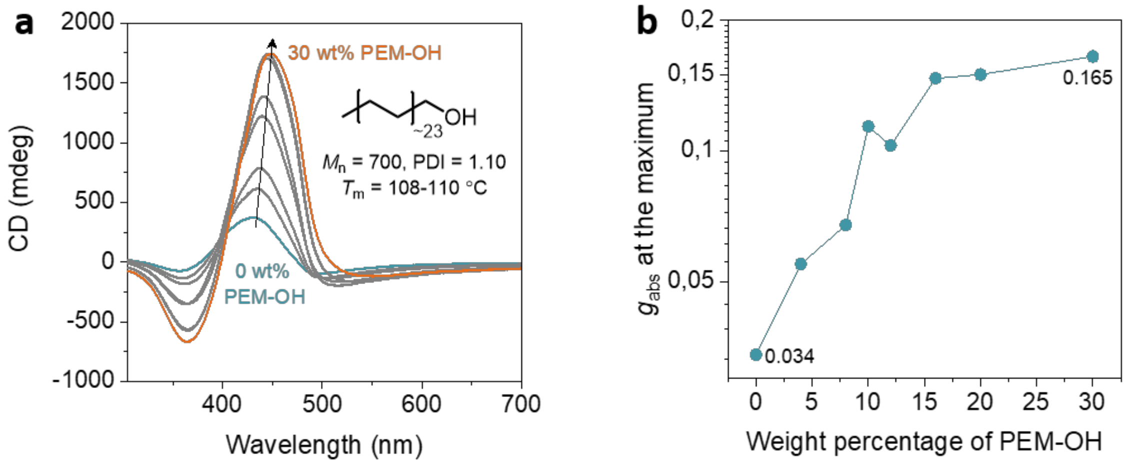

Figure S32: Effect of PEM-OH additive on chiroptical properties. a) Change in CD spectra on blending different weight percentages of PEM-OH with **(*S*,*S*)-PFAB**. The chemical structure of PEM-OH is shown in the inset along with its important properties. b) Evolution of dissymmetry factor for absorption (g_{abs}) at the maximum of CD effect as a function of weight percentage of PEM-OH loaded into the sample. All the samples were annealed at 150 °C for 15 minutes in a nitrogen glove box. The spectra are recorded at 20 °C. Film thickness ~60 nm.

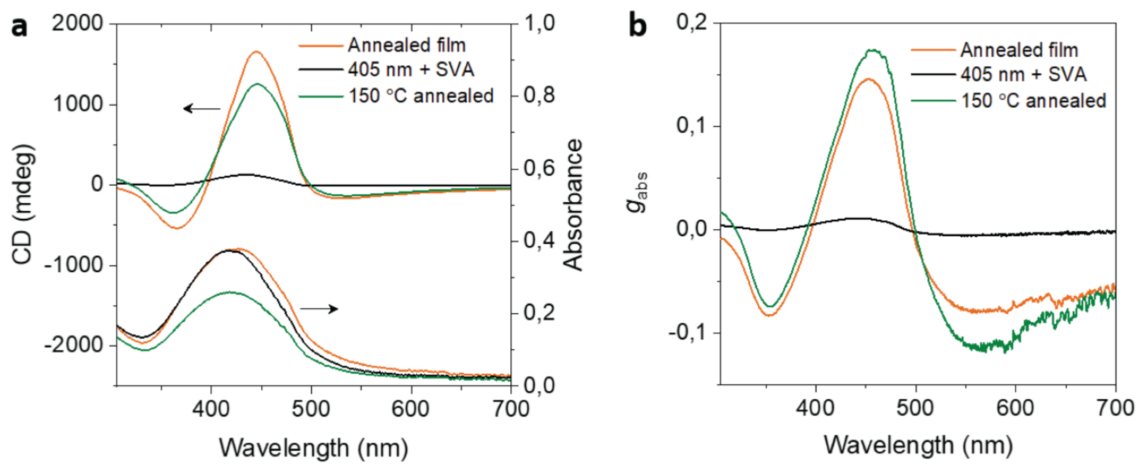

Figure S33: Effect of PEM-OH on photoswitchability in thin film. a) CD and UV-Vis spectra of 20wt% PEM-OH blended films of ***(S,S)*-PFAB**. b) Corresponding g_{abs} calculated from the CD and UV-Vis spectra from (a). Film thickness ~60 nm.

It can be noticed from the above graph (a) that on shining 405 nm light with SVA the CD effect shows a considerable decrease in intensity (by ~12 times). However, we do not see a significant drop is absorbance at the maximum, this might be due to the presence of PEM-OH. On thermal annealing (150 °C for 15 minutes), we observe that the CD effect is lower (1253 mdeg) compared to the freshly annealed film (1655 mdeg). However, this is mainly due to the significant drop in absorbance (by 0.12) on annealing. If we look at the normalized quantity (g_{abs}), we clearly notice that the g_{abs} is fully recovered on thermal annealing. The significant drop in absorbance on annealing is observed for many poly(fluorene)s and it is attributed to the reorganization of chains on thermal annealing. We anticipate that a similar process is taking place in our system as well.

For the *cis*→*trans* isomerization of azobenzene in (*S,S*)-PFAB we expect an increase in the absorption of the π – π* transition between 350 – 500 nm. However, the absorption for poly(fluorene) thin film is known to decrease on thermal annealing. Thus, due to these two opposing processes, it is hard to quantify the amount of back isomerization (*cis*→*trans*) of (*S,S*)-PFAB on thermal annealing.

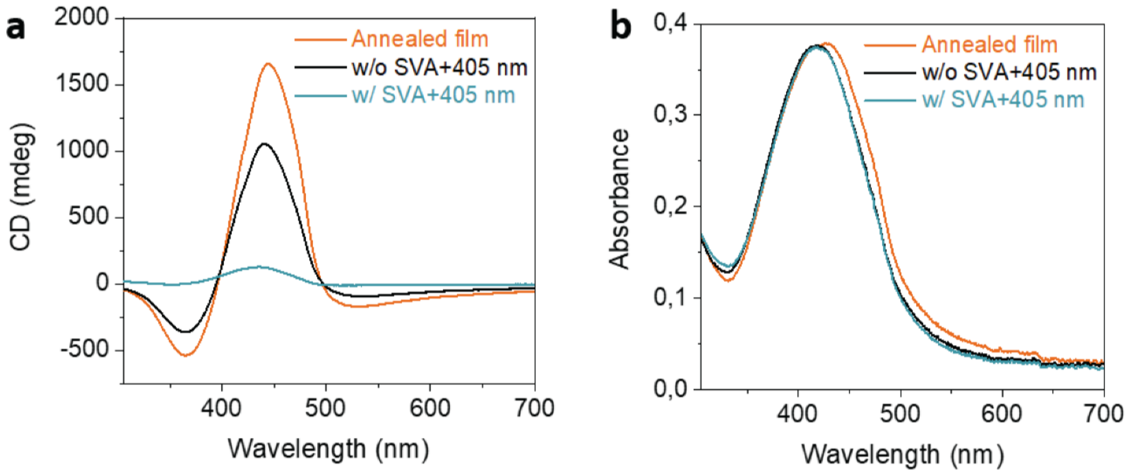

Figure S34: Effect of PEM-OH on *trans*→*cis* photoisomerization of **(*S*,*S*)-PFAB** with irradiation of 405 nm light. a) and b) CD and UV-Vis spectra, respectively under different conditions. Irradiation was done without depolarizer. Film thickness ~60 nm.

It can be clearly observed that without solvent vapor annealing (SVA), the CD effect decreases by only ~36% of its initial value, without much change in UV-Vis spectra. However, with SVA the CD effect drops to ~90% of its initial value. This experiment clearly suggests that presence of PEM-OH does not adversely affect the *trans*→*cis* photoisomerization. It is also to be noted that PEM-OH alone does not act as an efficient plasticizer to bring about *trans*→*cis* photoisomerization of **(*S*,*S*)-PFAB**.

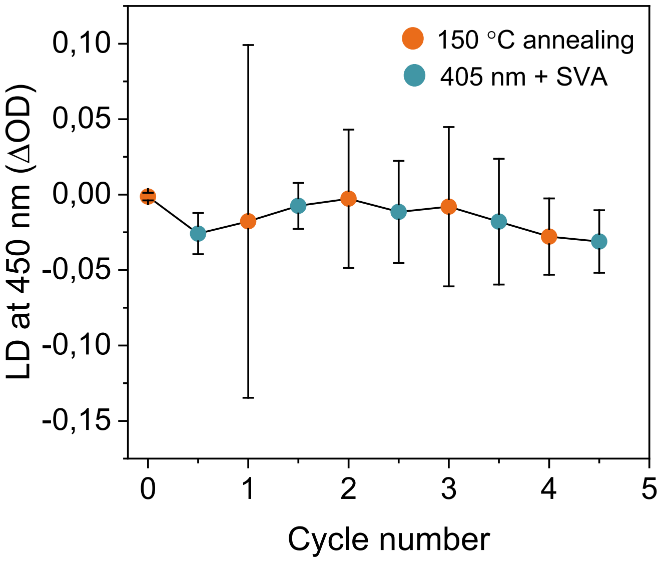

Figure S35: Variation of LD as a function of multiple irradiation (405 nm + SVA) and thermal annealing (150 °C) cycles for a 20wt% PEM-OH blended **(*S,S*)-PFAB** film. The irradiation of light was carried out with a depolarizer. The error bars represent the standard deviation calculated from three independent measurements. Film thickness ~60 nm.

It can be clearly observed that the mean of LD is very negligible, indicating that indeed the depolarizer aids in randomizing the residual polarization of incident light.

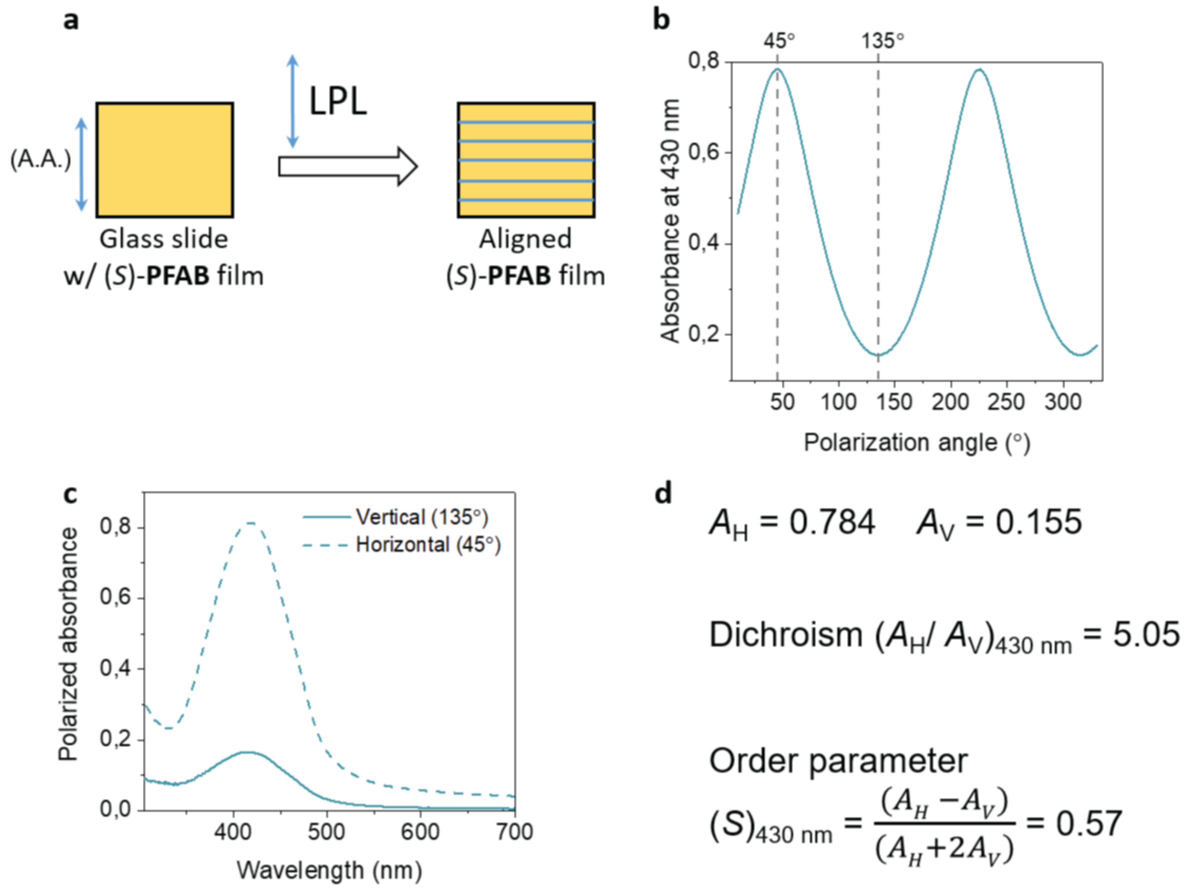

Figure S36: Effect of linearly polarized light on **(*S,S*)-PFAB** films. a) A schematic of the experiment in which a thin film of **(*S,S*)-PFAB** is irradiated with linearly polarized light (LPL). Here the direction on the left of the glass slide is an arbitrarily chosen axis (A.A.) with respect to which the direction of the LPL is varied. In this experiment, the direction of LPL and A.A. were parallel, as a result we anticipated that the polymer chains would reorient perpendicular to the direction of the LPL. All the polarized UV-vis spectra were recorded with the A.A. in a vertical position. b) Polarization angle dependence of absorbance at 430 nm. The angles 45° and 135° correspond to the horizontal (H) and vertical (V) direction, respectively. c) Full wavelength UV-vis spectra in horizontal and vertical positions. d) Calculation of the important parameters at 430 nm based on the spectra in (b&c). All the studies were carried out on **(*S,S*)-PFAB** film blended with 20wt% of PEM-OH. Irradiation was done with 405 nm LED in presence of SVA.

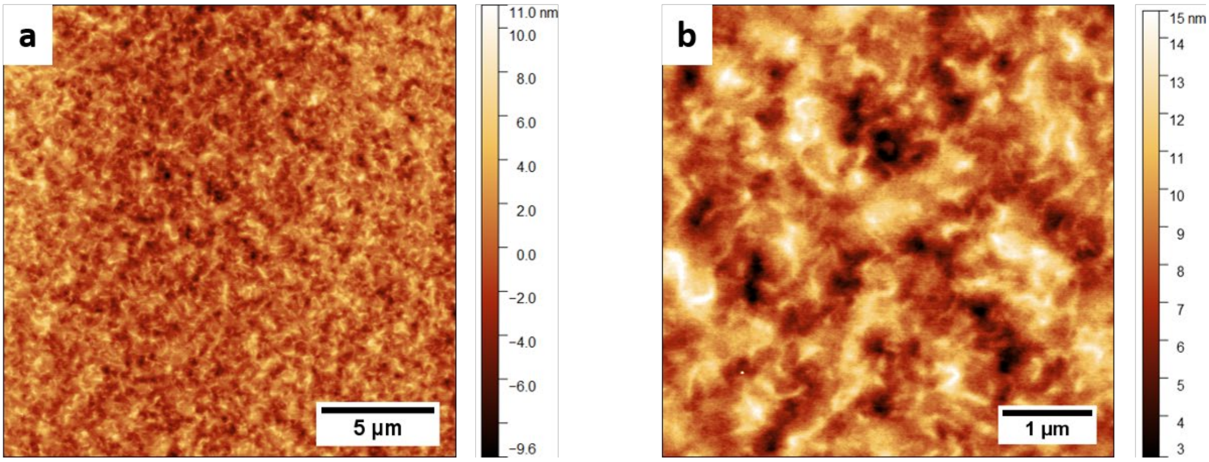

Figure S37: (a and b) AFM image of thermally annealed films of **(*S*,*S*)-PFAB** polymer without irradiation of light. AFM images at two different magnification are shown. Film thickness is ~70 nm.

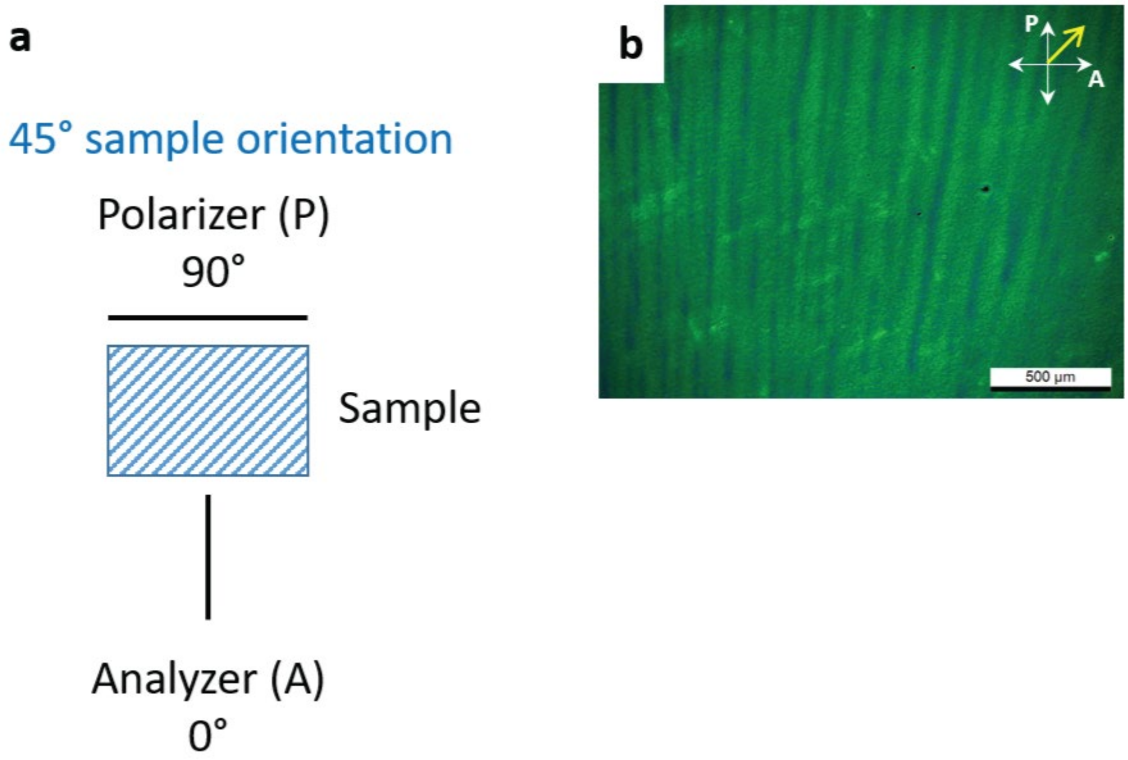

Figure S38: a) The experimental set-up used for POM analysis of aligned samples. b) A low magnification POM image of an aligned **(*S,S*)-PFAB** film. Film thickness is ~160 nm.

Experimental set-up for measuring *in-situ* kinetics of helicity change:

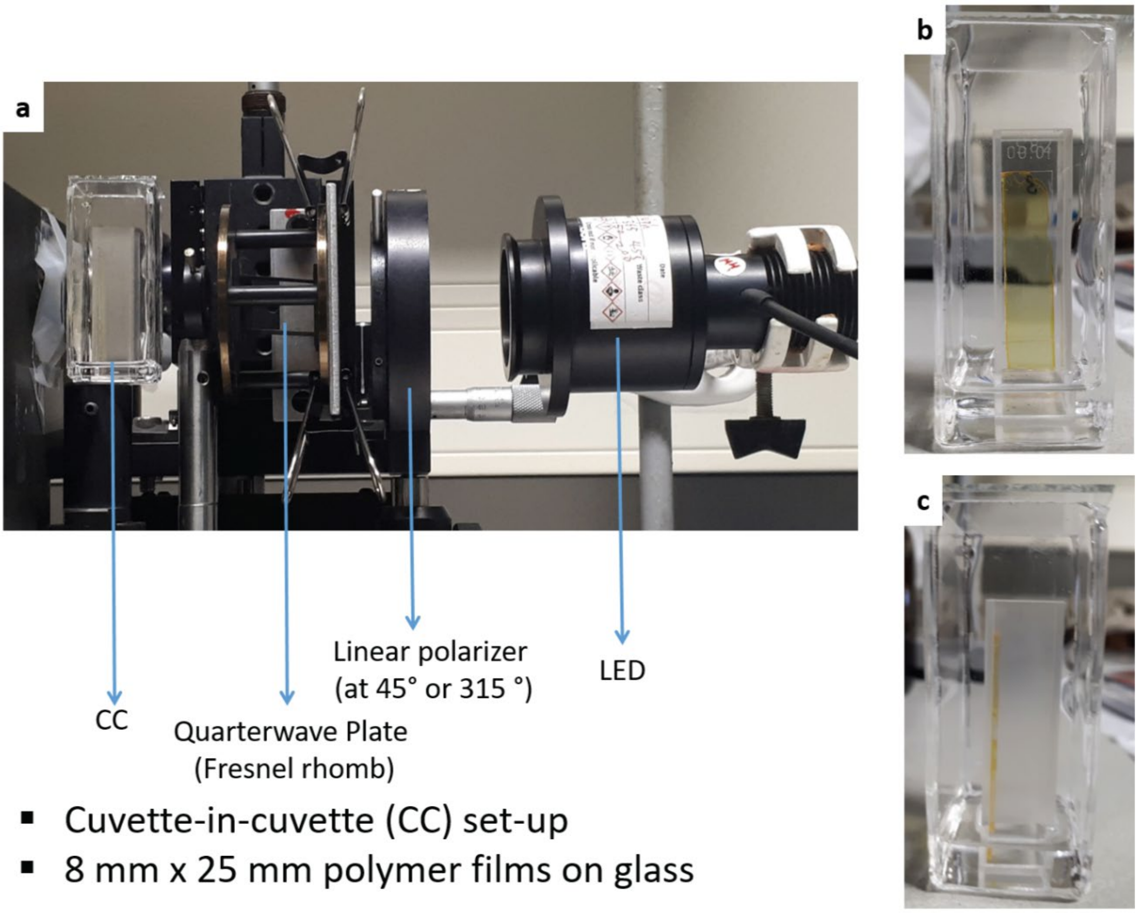

Figure S39: The experimental set-up used to measure the *in-situ* kinetics of helicity switching (*P*↔*M*). a) The experimental set-up of a modified circularly polarized light spectrometer with the irradiation part being shown. To the left of the image (not shown) there is a photoelastic modulator to record the polarization of light and a photon counting unit to quantify the polarization of light. b) and c) Front and side on views, respectively of the cuvette-in-cuvette set-up used for placing the sample.

The linear polarizer in combination with the Fresnel rhomb give CPL (either *R*- or *L*-). Since solvent vapor is critical for the action of light on the **(*S,S*)-PFAB** film, we have designed a cuvette-in-cuvette set-up to measure the kinetics in presence of solvent vapors. Here, a polymer film (~100 nm) was spin-coated on an 8 mm × 25 mm glass plate which fits inside a normal 10 mm path length cuvette. This normal cuvette (10 mm) holds the polymer coated glass slide and was inserted in another bigger square shaped quartz vial (25 mm × 25 mm base and 60 mm height) with an open top. The set-up was closed with glass plate. A certain level (~5 mm from bottom) of dichloromethane was maintained in the outer cuvette during the irradiation process to have a constant vapor pressure inside the inner cuvette.

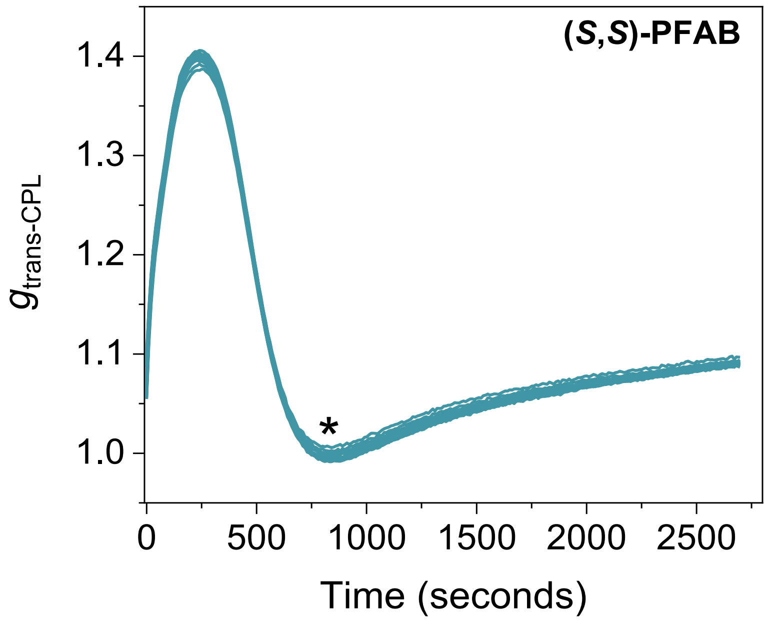

Figure S40: Kinetic profile of irradiating a thermally annealed ***(S,S)*-PFAB** film ((*P*)$_T$-helicity) with *L*-CPL. Film thickness was 130 nm. The experiment was carried out under SVA with irradiation by 455 nm LED. The dip in the profile marked with an asterisk mark (*) denotes a nematic like intermediate state.

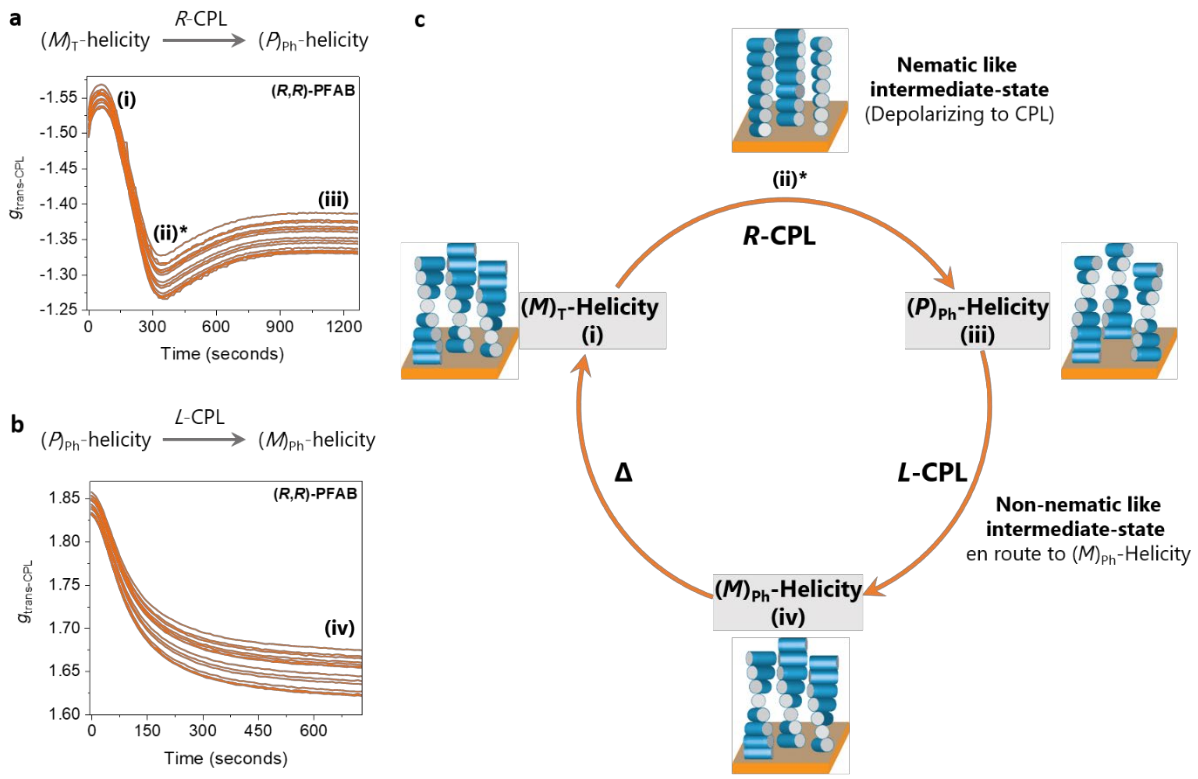

Figure S41: In-situ kinetics of ***(R,R)*-PFAB** films. a) Kinetic profile of a thermally annealed film of ***(R,R)*-PFAB** ((*M*)T-helicity) when continuously irradiated with *R*-CPL. b) The kinetic profile when the irradiation was switched to *L*-CPL. The experiments in (a) and (b) were performed sequentially on the same polymer film. The change in the sign of the $g_{\text{trans-CPL}}$ (Y-axis) in (a) and (b) is due to the switch in irradiation from *R*- to *L*-CPL. The various lines in (a) and (b) represent different monitoring wavelengths between 450-460 nm. c) A schematic illustration of the change in supramolecular helicity of polymer on irradiation of ***(R,R)*-PFAB** ((*M*)T-helicity) with *L*- and *R*-CPL. The (*M*)Ph-helicity on thermal annealing at 150 °C (denoted by 'Δ') reverts back to (*M*)T-helicity, thus completing a full cycle. The polymer chains are represented by rigid rods. The state marked with an asterisk (*) indicate an intermediate nematic like state. All the studies were carried out under SVA with irradiation by 455 nm LED (5 ± 1 mW/cm^2). ***(R,R)*-PFAB** polymer film thickness was 130 nm.

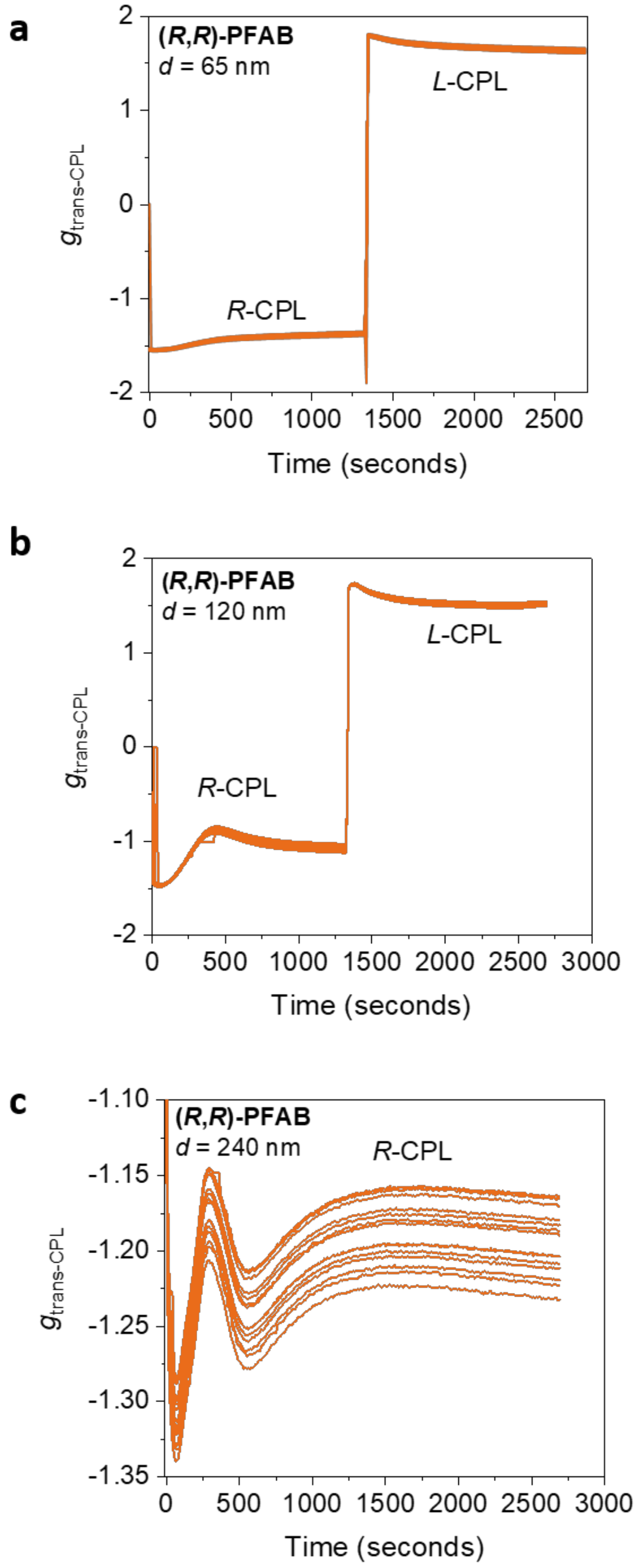

Figure S42: a), b) and c) Kinetic profiles of ***(R,R)*-PFAB** films at various thicknesses (*d*). All the films were thermally annealed to begin with ((*M*)$_T$-helicity) and irradiated first with *R*-CPL. The experiments were carried out under SVA with irradiation by 455 nm LED. An oscillatory nature of kinetic profile can be clearly observed.

4. References

[1] Stals, P. J. M.; Smulders, M. M. J.; Martín-Rapún, R.; Palmans, A. R. A.; Meijer, E. W. *Chem. Eur. J.* **15**, 2071-2080 (2009).

[2] Frisch, M. J.; Trucks, G. W.; Schlegel, H. B.; Scuseria, G. E.; Robb, M. A.; Cheeseman, J. R.; Scalmani, G.; Barone, V.; Mennucci, B.; Petersson, G. A.; Nakatsuji, H.; Caricato, M.; Li, X.; Hratchian, H. P.; Izmaylov, A. F.; Bloino, J.; Zheng, G.; Sonnenb, M. . H., J. J.; Brothers, E.; Kudin, K. N.; Staroverov, V. N.; Keith, T.; Kobayashi, R. ., Normand, J.; Raghavachari, K.; Rendell, A.; Burant, J. C.; Iyengar, S. S.; Tomasi, J. . & Cossi, M.; Rega, N.; Millam, J. M.; Klene, M.; Knox, J. E.; Cross, J. B.; Bakken, V.; Adamo, C.; Jaramillo, J.; Gomperts, R.; Stratmann, R. E.; Yazyev, O.; Austin, A. J.; Cammi, R.; Pomelli, C.; Ochterski, J. W.; Martin, R. L.; Morokuma, K.; Zakrzewski, V, G.; Voth, G. A.; Salvador, P.; Dannenberg, J. J.; Dapprich, S.; Daniels, A. D.; Farkas, O.; Foresman, J. B.; Ortiz, J. V.; Cioslowski, J. and Fox, D. J. Gaussian 09, Revision D.01, Gaussian, Inc, Wallingford CT, 2013.

[3] Becke, A. D. Density-functional exchange-energy approximation with correct asymptotic behavior. *Phys. Rev. A* **38,** 3098–3100 (1988).

[4] Lee, C., Yang, W. & Parr, R. G. Development of the Colle-Salvetti correlation-energy formula into a functional of the electron density. *Phys. Rev. B* **37,** 785–789 (1988).

[5] Abbel, R., Schenning, A. P. H. J. & Meijer, E. W. Molecular weight optimum in the mesoscopic order of chiral fluorene (Co)polymer films. *Macromolecules* **41,** 7497–7504 (2008).

[6] Sivamani, J., Balasaravanan, R., Duraimurugan, K. & Siva, A. Synthesis, characterization and photophysical studies of self-assembled azo biphenyl urea derivatives. *Photochem. Photobiol. Sci.* **15,** 211–218 (2016).

[7] Kulkarni, C., Meskers, S. C. J., Palmans, A. R. A. & Meijer, E. W. Amplifying Chiroptical Properties of Conjugated Polymer Thin film Using an Achiral Additive. *Macromolecules* **51,** 5883–5890 (2018).

[8] Kulkarni, C., Di Nuzzo, D., Meijer, E. W. & Meskers, S. C. J. Pitch and Handedness of the Cholesteric Order in Films of a Chiral Alternating Fluorene Copolymer. *J. Phys. Chem. B* **121,** 11520–11527 (2017).

[9] Good, R. H. & Karali, a. Transmission of light through a slab of cholesteric liquid crystal. *J. Opt. Soc. Am. A* **11,** 2145 (1994).
